# Efficacy and Safety of CAR-T Cell Products Axicabtagene Ciloleucel, Tisagenlecleucel, and Lisocabtagene Maraleucel for the Treatment of Hematologic Malignancies: A Systematic Review and Meta-Analysis

**DOI:** 10.3389/fonc.2021.698607

**Published:** 2021-07-26

**Authors:** Jun Meng, XiaoQin Wu, Zhen Sun, RenDe Xun, MengSi Liu, Rui Hu, JianChao Huang

**Affiliations:** ^1^ Molecular Genetics Laboratory, Suining Central Hospital, Suining, China; ^2^ Department of Neurosurgery, The First Affiliated Hospital, University of South China, Hengyang, China; ^3^ Hengyang Medical College, University of South China, Hengyang, China

**Keywords:** chimeric antigen receptor T-cell product, CAR-T cell therapy, immunotherapy, lymphoma, leukemia, hematologic malignancy, efficacy, safety

## Abstract

**Background:**

Currently, three chimeric antigen receptor (CAR)-T cell products axicabtagene ciloleucel, tisagenlecleucel, and lisocabtagene maraleucel have been approved by the U.S. Food and Drug Administration for the treatment of large B cell lymphoma, which provide a novel and promising choice for patients with relapsed or refractory to traditional anti-tumor treatments. Thus, it is pertinent to describe the efficacy and safety profile of the three products available by summarizing the current evidence.

**Methods:**

Two reviewers independently searched the Embase, PubMed, Web of Science, and Cochrane Library, to identify studies related to the use of the three CAR-T cell products for treating hematologic malignancies published up to October 5, 2020. We pooled the overall response rate, complete response rate, cytokine release syndrome, and immune effector cell-associated neurotoxicity syndrome of three products, and then performed subgroup analysis based on the type of product and type of tumor.

**Results:**

Thirty-three studies involving 2,172 patients were included in the analysis. All three products showed promising results in patients with different pathological subtypes and clinical characteristics that included those who did not meet the eligibility criteria of licensing trials, with overall response rates of nearly 70% or above and complete response rates of more than 50%. However, high rates of severe immune effector cell-associated neurotoxicity syndrome in patients undergoing axicabtagene ciloleucel treatment and life-threatening cytokine release syndrome in patients with leukemia undergoing tisagenlecleucel treatment required special attention in practice (31%; 95% CI: 0.27–0.35 and 55%; 95% CI: 0.45–0.64, respectively). Moreover, lisocabtagene maraleucel that showed a favorable efficacy and safety in the licensing trial lacked corresponding real-world data.

**Conclusion:**

Both axicabtagene ciloleucel and tisagenlecleucel showed considerable efficacy in practice, but need special attention with respect to life-threatening toxicity that can occur in certain situations. Lisocabtagene maraleucel demonstrated excellent efficacy and safety profiles in the licensing trial, but lacked corresponding real-world data. Additional data on the three products are needed in rare histological subtypes to benefit a broader patient population.

## Introduction

First conceptualized in the late 1980s, chimeric antigen receptor (CAR)-T cell therapy has developed rapidly over the decade and is considered one of the most promising treatments for hematologic malignancies (1). CAR-T cell therapy involves injecting of genetically modified autologous or allogeneic T cells into the patient to specifically target patient’s tumor cells (2). The efficacy of CAR T-cell therapy appears considerably better than that of traditional chemotherapy and autologous/allogeneic stem cell transplant in the setting of relapsed/refractory disease, however, it is associated with potentially fatal side effects such as cytokine release syndrome (CRS) and immune effector cell-associated neurotoxicity syndrome (ICANS) (3).

A CAR consists of antigen-binding domains (most commonly, a single-chain variable fragment), transmembrane domains, signaling domains, and additional co-stimulatory domains (2, 4). To date, CAR-T cells have progressed from the first generation to the fourth generation. The main difference between the first- and second-generation CAR-T cells is the incorporation of co-stimulatory endodomain. The anti-tumor activity of the first-generation CARs is disappointing because it only contains CD3ζ signaling domain, while the second-generation CARs possess one co-stimulatory endodomain (CD28, 4-1BB, or OX40) incorporated with CD3ζ, which can effectively promote T cell activation and prevent apoptosis. The third-generation CAR-T cells contain multiple co-stimulatory domains, and the fourth-generation CAR-T cells, also called TRUCKs, need additional clinical data to demonstrate their safety and efficacy (4, 5). Currently, five second-generation CAR-T cell products, axicabtagene ciloleucel (axi-cel), tisagenlecleucel (tisa-cel), lisocabtagene maraleucel (liso-cel), brexucabtagene autoleucel, and idecabtagene vicleucel have been approved by the U.S. Food and Drug Administration due to their prominent efficacy^1^; axi-cel, tisa-cel, and liso-cel have been approved for similar indications of large B cell lymphoma. All three products use anti-CD19 single-chain variable fragment to recognize and target tumor antigens. Both tisa-cel and liso-cel utilize a 4-1BB co-stimulatory domain fused to CD3ζ signaling domain, and axi-cel utilizes a CD28 co-stimulatory domain fused to CD3ζ signaling domain for full T-cell activation. In addition, liso-cel is administered as a sequential infusion of two components (CD8⁺ and CD4⁺ CAR⁺ T cells) at equal target doses, whereas both axi-cel and tisa-cel are generated from bulk T cells; however, the proportion of these cells differ among different patients. Differences in these key elements lead to different expansion, persistence, and cytotoxicity *in vivo* of the three products, and it is still not concluded about which structure is better (6) (**Table 1**).

**Table 1:** CAR T-cell product composition comparisons.

	Axicabtagene ciloleucel	Tisagenlecleucel	Lisocabtagene maraleucel
Target Antigen (scFv)	CD19	CD19	CD19
Transmembrane domain	CD28	CD8-*α*	CD28
Co-stimulation domain	CD28	4-1BB	4-1BB
T-cell manufacturing	Unspecified	Unspecified	1:1 CD4:CD8
Signaling domain	CD3ζ	CD3ζ	CD3ζ
Indications	Adult patients with relapsed or refractory (r/r) large B-cell lymphoma after two or more lines of systemic therapy.	1. Patients up to 25 years of age with r/r B-cell ALL 2. Adult patients with r/r large B-cell lymphoma after two or more lines of systemic therapy.	Adult patients with relapsed or refractory (r/r) large B-cell lymphoma after two or more lines of systemic therapy.

Meta-analysis and systematic reviews on the safety and efficacy of CAR-T cell therapy for hematologic and solid malignancies have been published, but a comprehensive evaluation of the three currently marketed CAR-T cell products is lacking (7–15). In real-world clinical setting, most patients receive only these approved products rather than the experimental CAR-T cells that were mainly focused in the previous studies. In addition, the pivotal trials that supported the approval of the three CAR-T cell products were limited by the strict inclusion and exclusion criteria, and therefore the characteristics of the study patients were different from those in the real-world. For example, more than half of the patients with new diffuse large B cell lymphoma (DLBCL) are older than 65 years and show a worse prognosis than younger patients; however, most patients included in the pivotal trials were younger than 65 years; thus, it may limit the generalizability of trial findings (16–19). Therefore, it is necessary to conduct a systematic study including more data to establish the performance and differences of the three products.

Our study aims to analyze the risks and benefits associated with the three CAR-T cell products in the treatment of malignant tumors through systematically summarizing the existing relevant literature and data, which will further assist clinicians in choosing more appropriate products and preventing side effects in patients.

## Methods

The present systematic review and meta-analysis followed the Preferred Reporting Items for Systematic Reviews and Meta-analyses (PRISMA) statement, and the protocol was registered in the International Prospective Register of Systematic Reviews (CRD42020197902) (20).

### Eligibility Criteria

The types of study involved all phases of clinical trials, including randomized or non-randomized controlled trials and single-arm studies. The study participants were all patients with hematologic malignancies treated with either of the three CAR-T cell products (axi-cel, tisa-cel, and liso-cel). The efficacy outcomes of interest were complete response rate (CRR) and overall response rate (ORR) defined by the combined rate of complete and partial responses. Safety outcomes of interest were severe CRS and ICANS defined by grade 3 or higher. Case series involving less than four patients, conference abstracts, reviews, editorials, commentary, animal experiments, unpublished gray literature, and other literature with unavailable study data were excluded.

### Search Strategies

We systematically searched the Embase, PubMed, Web of Science, and Cochrane Library to identify relevant articles by subject words combined with free words. Databases were searched on October 5, 2020. Additionally, we reviewed the reference lists of related reviews and included articles, and there was no language limit: (1) Subject words: Neoplasms; Malignant Neoplasm; Free words: Neoplasia; Neoplasias; Neoplasm; Tumors; Tumor; Cancer; Cancers; Malignancy; Malignancies; Malignant Neoplasms; Malignant Neoplasm; Neoplasm, Malignant; Neoplasms, Malignant; Benign Neoplasm; Neoplasms, Benign; Malignant Tumor; Neoplasm, Benign; (2) Subject word: Axicabtagene ciloleucel; Free words: Yescarta; KTE-C19; (3) Subject word: Tisagenlecleucel; Free words: KYMRIAH; CTL019; (4) Subject word: Lisocabtagene maraleucel; Free words: jcar 017; jcar 17; The complete search strategy for each database is available in the Appendix.

### Data Extraction and Quality Assessment

According to the eligibility criteria, two reviewers independently reviewed and screened the literature and cross-checked the included literature. The reasons for exclusion were recorded, and any discrepancies were resolved by consensus of all reviewers. We collected data of eligible literature including first author, year of publication, number of patients, age, gender, type of CAR-T cell, type of tumor, scales of toxicity and response, CRS, ICANS, and response outcomes. The Newcastle-Ottawa scale (NOS) was used to assess the quality of the included literature, including selection, comparability, and outcome.

### Statistical Analysis

In this study, data analysis was performed using the Stata 14.0 software. Dichotomous data (ORR, CRR, CRS, and ICANS) with 95% confidence intervals (95% CIs) were analyzed to estimate the efficacy and adverse effects of the three CAR-T cells; P ≤0.05 was considered statistically significant. Due to rapid deterioration of the patients with lymphoma, majority of CAR-T cell therapy clinical trials are single-arm studies. Considering the lack of a control group to balance the differences in eligibility criteria and subsequent intervention methods of each study, we used a random-effects model for all data synthesis to better reflect real-world conditions, and fixed-effects model was used simultaneously for sensitivity analysis. Moreover, we assessed the heterogeneity of the included studies by Cochran’s Q-test and Higgins’ I^2^ statistic. The heterogeneity was considered significant at P <0.1 and I^2^ >75%; the sources of heterogeneity were identified by reviewing the patient characteristics, type of CAR-T product, or endpoint assessment scale of the included studies. Only when heterogeneity remained following the above steps, we used the method of excluding one study at a time (21). Subgroup analyses were performed to investigate the efficacy and safety according to the type of product and type of tumor. We assessed publication bias by funnel plots and confirmed by Egger’s and Begg’s tests. When arising a symmetrical inverted funnel shape, and Egger’s and Begg’s test yielded P-values greater than 0.5, we consider no publication bias.

## Results

### Study Characteristics

**Figure 1:**
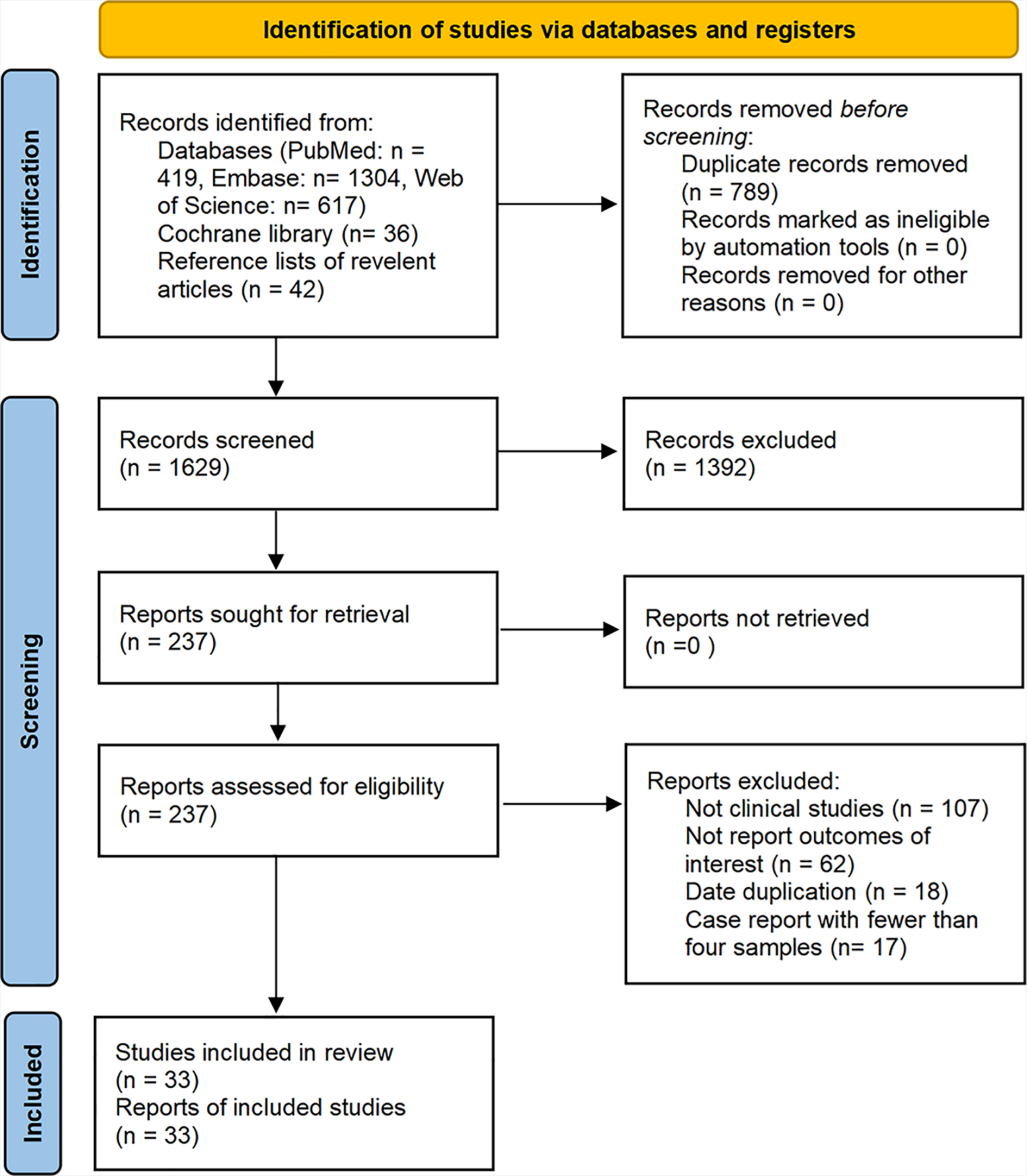
Flow diagram of the study select process.

The database search resulted in identification of a total of 2,418 articles published on studies related to the treatment of malignant tumors with axi-cel, tisa-cel, or liso-cel, of which 33 studies met our eligibility criteria and were included in the analysis after de-duplication and screening title, abstract, and full-text. These studies included eighteen axi-cel, nine tisa-cel, one liso-cel, and five studies with both axi-cel and tisa-cel (**Figure 1**).

A total of 2,172 patients were analyzed from the selected studies, including 1,352 (62.2%) patients with DLBCL, 192 (8.8%) patients with follicular lymphoma (FL) or transformed follicular lymphoma (tFL), 95 (4.3%) patients with primary mediastinal B-cell lymphoma (PMBCL), 70 (3.2%) patients with high-grade B cell lymphoma (HGBCL), 140 (6.4%) patients with acute lymphoblastic leukemia (ALL), 14 (0.6%) patients with chronic lymphocytic leukemia (CLL), 10 (0.5%) patients with multiple myeloma (MM), 6 patients with transformed marginal zone lymphoma (TMZL), 11 patients with Richter’s syndrome (RS), and 282 (13.0%) patients with unidentified tumor types. Among all patients, 1,718 (79.1%) were evaluated for response, 1,860 (85.6%) were evaluated for cytokine release syndrome (CRS), and 2,079 (95.7%) were evaluated for ICANS.

There were 14 studies with median age of the patients <60 years, 15 studies with median age ≥60 years, 1 study with mean age ≥60, and 3 studies did not report age of the patients. The studies by Sermer et al. and Wudhikarn et al. included patients treated in the same institution for a similar period, so the data of the patient had a large overlap; however, Sermer et al. reported only efficacy data, while Wudhikarn et al. reported only adverse effects, so data synthesis was not affected (22, 23). Moreover, the studies by Frey et al. (30 samples) and by Maude et al. (35 samples) included five patients with ALL from the same trial (ClinicalTrials.gov number: NCT02030847); however, considering the proportion of overlapping patients was small, no study was omitted (24, 25). Sensitivity analysis was performed to test for stability in subgroup analysis of patients with ALL. Notably, all patients included in the pooled analysis actually received CAR-T cell infusion, and those patients were excluded who were intent to receive CAR-T cell administration but finally discontinued. The detailed characteristics of the included studies are shown in **Table 2**.

**Table 2:** Characteristics of included studies.

First Author	Year	No.	Median age (range)-year	Histological type	CAR-T type	Efficacy evaluation	Scale	Toxicity evaluation (grade ≥3)	Scale (CRS/ICANS)	Ref
Schuster	2018	111	56 (22–76)	DLBCL	tisa-cel	CR: 37/93	Lugano	CRS: 24/111	Penn/CTCAE 4.03, MDRA 20.1	(17)
						PR: 11/93		ICANS: 13/111		
Schubert	2020	21	52 (20–68)	16 DLBCL, 3 PMBCL, 1 DHL, 1 tFL	axi-cel	CR: 9/21	Lugano	CRS: 0/21	ASTCT/ASTCT	(26)
						PR: 10/21		ICANS: 6/21		
Pinnix	2020	124	60 (18–85)	95 DLBCL, 20 tFL, 9 PMBCL	axi-cel	CR: 60/124	Lugano	CRS: 11/124	ASTCT, CARTOX/ASTCT, CARTOX	(27)
						PR: 36/124		ICANS: 49/124		
Nastoupil	2020	298	60 (21–83)	203 DLBCL, 76 tFL, 19 PMBCL	axi-cel	CR: 175/275	Lugano	CRS: 19/275	CARTOX, Lee/CARTOX, CTCAE 4.03	(28)
						PR: 50/175		ICANS: 85/275		
Neelapu	2017	101	58 (23–76)	77 DLBCL, 16 tFL, 8 PMBCL	axi-cel	CR: 55/101	IWGRC	CRS: 13/101	Lee/CTCAE 4.03	(19)
						PR: 28/101		ICANS: 28/101		
Locke	2017	7	46 (29–69)	DLBCL	axi-cel	CR: 4/7	IWGRC	CRS: 1/7	Lee/CTCAE 4.03	(29)
						PR: 1/7		ICANS: 4/7		
Jain	2019	4	56 (38–66)	DLBCL	axi-cel	CR: 2/4	NP	CRS: 0/4	NP/NP	(30)
						PR: 1/4		ICANS: 0/4		
Abbasi	2020	10	66 (55–77)	DLBCL	axi-cel	CR: 8/10	NP	CRS: 1/10	ASTCT/ASTCT	(31)
						PR: 0/10		ICANS: 3/10		
Garfall	2018	10	61 (48–68)	MM	tisa-cel	CR: 6/10	IMWGRC	CRS: 0/10	NP/NP	(32)
						PR: 2/10		ICANS: 0/10		
Maude	2018	75	11 (3–23)	ALL	tisa-cel	CR: 61/75	Independent scale	CRS: 35/75	Penn/CTCAE 4.03	(33)
						PR: 0/75		ICANS: 10/75		
Maude	2014	30	14 (5–60)	ALL	tisa-cel	CR: 27/30	Independent scale	CRS: 8/30	Independent scale/NP	(24)
						PR: 0/30		ICANS: NP		
Schuster	2017	28	58 (25–77)	14 DLBCL	tisa-cel	CR: 16/28	1999 IWGRC	CRS: 5/28	Penn/NP	(34)
				14 FL		PR: 2/28		ICANS: 3/28		
Frigault	2019	8	50 (17–79)	5 DLBCL, 2 HGBCL, 1 PMBCL	tisa-cel	CR: 2/8	NP	CRS: 0/8	Lee, ASTCT/Lee, ASTCT	(35)
						PR: 2/8		ICANS: 0/8		
Sim	2019	11	NP	8 DLBCL, 3 tFL,	axi-cel	CR: 5/11	Lugano	CRS: 1/11	CTCAE 5.0/CTCAE 5.0	(36)
						PR: 4/11		ICANS: 3/11		
Porter	2015	14	66 (51–78)	CLL	tisa-cel	CR: 4/14	IWG on CLL RC	CRS: 7/14	Penn/CTCAE 3.0	(37)
						PR: 4/14		ICANS: 1/14		
Shah	2018	7	NP	3 DLBCL, 4 FL	tisa-cel	CR: 3/7	Lugano	CRS: NP	NP/NP	(38)
						PR: 2/7		ICANS: NP		
Wright	2020	31	NP	26 DLBCL, 5 tFL	18 axi-cel, 13 tisa-cel	CR: 11/27	Lugano	CRS: 6/31	Penn/NP	(39)
						PR: 3/27		ICANS: 4/31		
Jacobson	2020	122	62 (21–79)	57 DLBCL, 33 tFL, 17 HGBCL, 8 PMBCL, 5 TMZL, 2 RS	axi-cel	CR: 61/122	Lugano	CRS: 19/122	Lee/CTCAE 4.03	(40)
						PR: 24/122		ICANS: 43/122		
Abramson	2019	268	63 (18–86)	206 DLBCL, 33 HGBCL, 14 PMBCL, 2FL3B	liso-cel	CR: 135/255	Lugano	CRS: 6/268	Lee/CTCAE 4.03	(16)
						PR: 51/255		ICANS: 27/268		
Fehse	2019	10	56 (24–79)	7 DLBCL, 3 PMBCL	axi-cel	CR: 2/10	NP	CRS: 2/10	ASTCT/ASTCT	(41)
						PR: 5/10		ICANS: 1/10		
Gupta	2019	78	60+–13※	DLBCL	69 axi-cel, 9 tisa-cel	CR+PR: 43/78*	NP	CRS: 10/78	CTCAE 5.0, Lee/CTCAE 5.0	(42)
								ICANS: 22/78		
Korell	2020	25	54 (20–68)	24 DLBCL, 1 PMBCL	axi-cel	CR: 9/25	Lugano	CRS: NP	NP/NP	(43)
						PR: 10/25		ICANS: NP		
Frey	2019	35	34 (21–70)	ALL	tisa-cel	CR: 24/35	Independent scale	CRS: 25/35	Penn/CTCAE 4.03	(25)
						PR: 0/35		ICANS: 2/35		
Sesques	2020	61	59 (27–75)	38 DLBCL, 18 PMBCL, 4 tFL, 1 TMZL	28 axi-cel, 33 tisa-cel	CR: 28/61	Lugano	CRS: 5/61	ASTCT/ASTCT	(44)
						PR: 9/61		ICANS: 6/61		
Holtzman	2020	45	60 (26–75)	35 DLBCL, 3 PMBCL, 7 tFL	axi-cel	CR: 22/45	NP	CRS: NP	NP/CTCAE 4.03	(45)
						PR: NP		ICANS: 18/45		
Strati	2020	100	60 (18–85)	LBCL (Including 77 DLBCL)	axi-cel	CR: NP	Lugano	CRS: 9/100	CARTOX/CARTOX	(46)
						PR: NP		ICANS:41/100		
Faramand	2020	75	63 (23–79	50 DLBCL, 25 Transformed Indolent lymphomas	axi-cel	CR: 36/68	Lugano	CRS: 12/75	ASTCT/CARTOX, ASTCT, CTCAE v4.03	(47)
						PR: 29/68		ICANS: 23/75		
Kittai	2020	9	64 (40–77)	RS	axi-cel	CR: 8/8	Lugano	CRS: 1/9	ASTCT/ASTCT	(48)
						PR: 5/8		ICANS: 3/9		
Deng	2020	24	58 (24–74)	16 DLBCL, 6 tFL, 2 PMBCL	axi-cel	CR: NP	NP	CRS: 4/24	NP/NP	(49)
						PR: NP		ICANS: 12/24		
Dean	2020	96	64 (19–79)	47 DLBCL, 15 HGBCL, 5 PMBCL, 29 NP	axi-cel	CR: 74/96	NP	CRS: 9/96	Lee/CTCAE 4.03	(50)
						PR: 63/96		ICANS: 28/96		
Sermer	2020	69	63 (19–85)	DLBCL	47 axi-cel, 22 tisa-cel	CR: 50/69	Lugano	CRS: NP	NP/NP	(22)
						PR: 36/69		ICANS: NP		
Wudhikarn	2020	60	63 (20–86)	DLBCL	43 axi-cel, 17 tisa-cel	CR: NP	NP	CRS: 7/60	NP/NP	(23)
						PR: NP		ICANS: 13/60		
Rubin	2020	204	60+–12^※^	Inexact^#^	axi-cel	CR: NP	NP	CRS: NP	NP/CTCAE 4.03	(51)
						PR: NP		ICANS: 51/204		

CR, complete response; PR, partial response; CRS, cytokine release syndrome; ICANS, immune effector cell-associated neurotoxicity syndrome; DLBCL, diffuse large B cell lymphoma; FL/tFL, follicular lymphoma or transformed follicular; PMBCL, primary mediastinal B-cell lymphoma; HGBCL, high-grade B cell lymphoma; ALL, acute lymphoblastic leukemia; CLL, chronic lymphocytic leukemia; CAR-T, chimeric antigen receptor T; TMZL, transformed marginal zone lymphoma; MM, multiple myeloma; NP, not provided; ref, reference; MDRA, Medical Dictionary for Regulatory Activities, version; CTCAE, Common Terminology Criteria for Adverse Events; RS, Richter’ s syndrome; ASTCT, American Society for Transplantation and Cellular Therapy criteria; CARTOX, CAR-T-cell-therapy-associated TOXicity; IWGRC, International Working Group Response Criteria; IMWGRC, International Myeloma Working Group response criteria; IWG on CLL RC, International Workshop Group on CLL response criteria; Independent scale, the institution used their own criteria instead of international criteria, which can be found in original text. *No separate CR and PR numbers were provided ※Mean ± standard deviation. ^#^Patients with aggressive (e.g., diffuse large B-cell lymphoma, primary mediastinal B-cell lymphoma) or indolent (e.g., follicular lymphoma, marginal zone lymphoma) histologic subtype.

All studies were independently assessed for quality using NOS (cohort studies). Since all included studies were single-arm studies, the selection of the non-exposed cohort of NOS was not applicable. Overall study quality was rated as moderate to high as shown in **Table S1**.

### Meta-Analysis of Overall Efficacy of the CAR-T Cell Products

**Figure 2:**
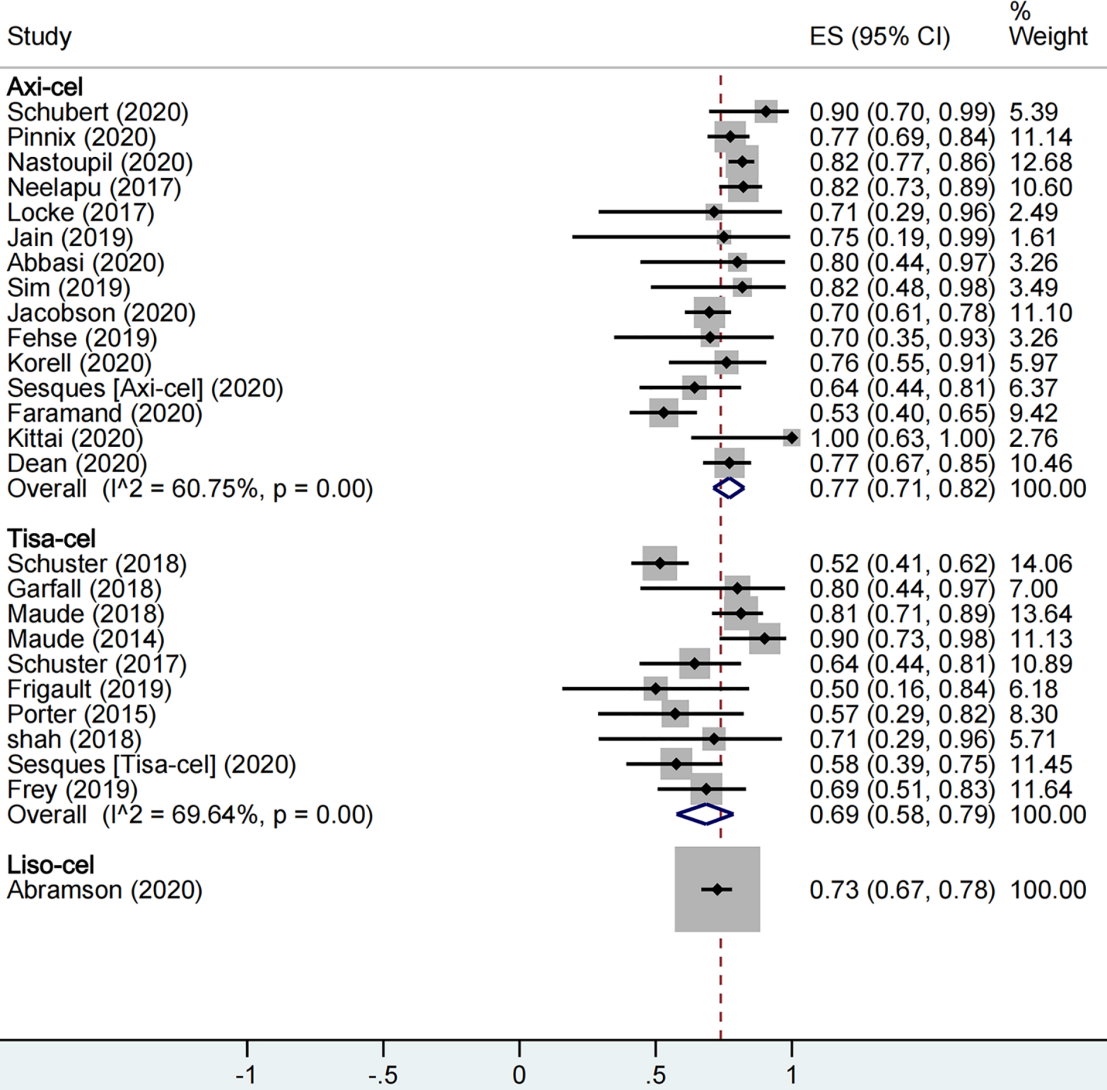
The forest plot of total overall response rate of each product.

**Figure 3:**
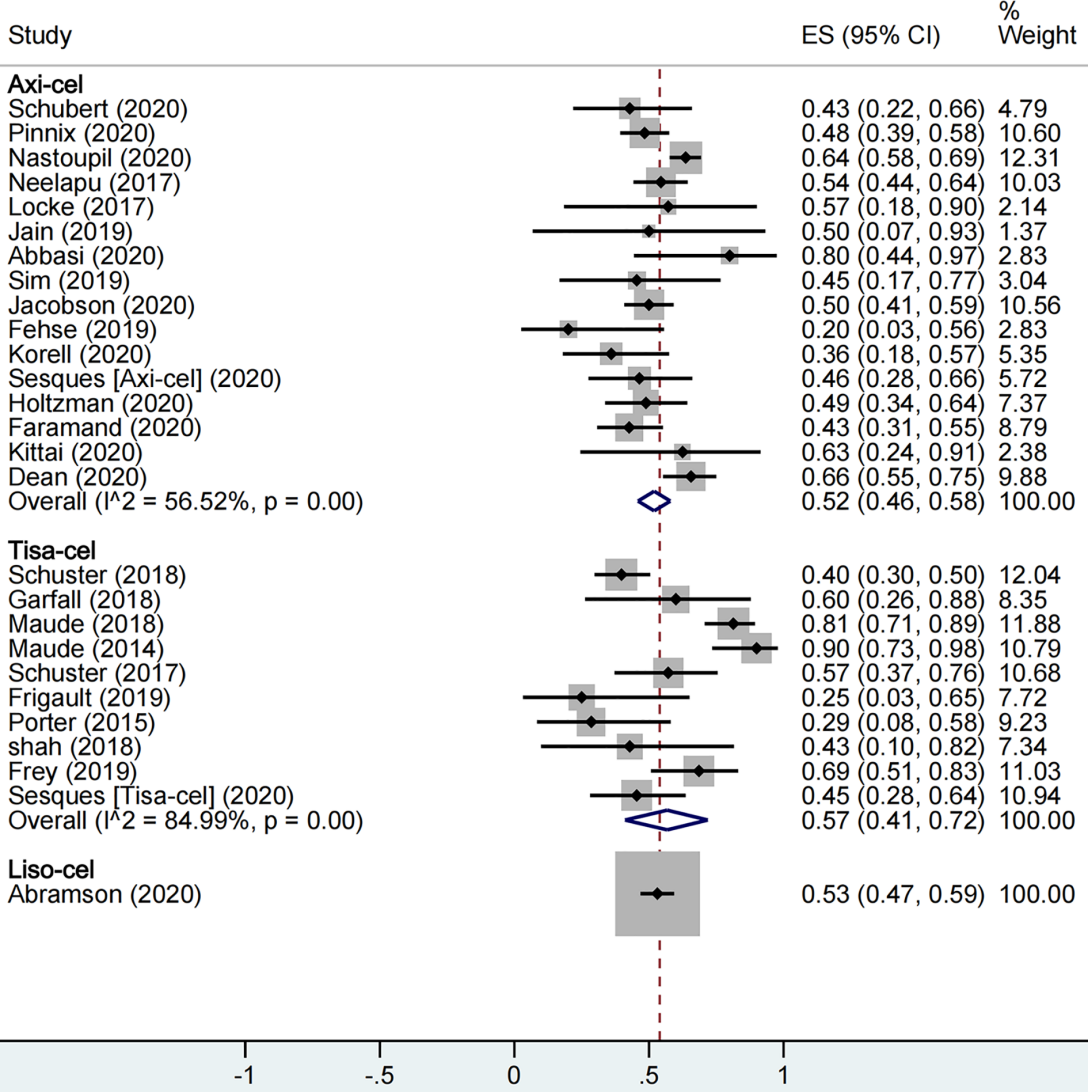
The forest plot of total complete response rate of each product.

A total of 1,673 patients were included for ORR evaluation. ORR was calculated as 73% (95% CI: 0.68–0.77; I^2^ = 70.9%, P < 0.01) for all patients, and axi-cel, tisa-cel, and liso-cel groups showed individual response rates of 77% (95% CI: 0.71–0.82; I^2^ = 60.8%, P < 0.01), 69% (95% CI: 0.58–0.79; I^2^ = 69.6%, P < 0.01), and 73% (95% CI: 0.67–0.78), respectively (**Figure 2**). A total of 1,640 patients were included for CRR evaluation. CRR was calculated as 54% (95% CI: 0.48–0.59; I^2^ = 73.0%, P < 0.01) for all patients, and it was estimated as 52% (95% CI: 0.46–0.58; I^2^ = 56.5%, P < 0.01), 57% (95% CI: 0.41–0.72; I^2^ = 85.0%, P < 0.01), and 53% (95% CI: 0.47–0.59) in axi-cel, tisa-cel, and liso-cel groups, respectively (**Figure 3**).

Among all, tisa-cel group showed significant heterogeneity. Therefore, we divided the group into lymphoma group and ALL group for subgroup analysis considering that the indications of tisa-cel included lymphoma and ALL. The respective ORR and CRR were estimated as 57% (95% CI: 0.50–0.65; I^2^ = 0.0%, P = 0.50) and 44% (95% CI: 0.36–0.52; I^2^ = 0.0%, P = 0.47) in the lymphoma group, whereas CRR was estimated as 81% (95% CI: 0.69–0.90; I^2^ = 55.7%, P = 0.10) in ALL group, suggesting this indication as the source of heterogeneity in tisa-cel study group.

### Meta-Analysis of Overall Safety of the CAR-T Cell Products

**Figure 4:**
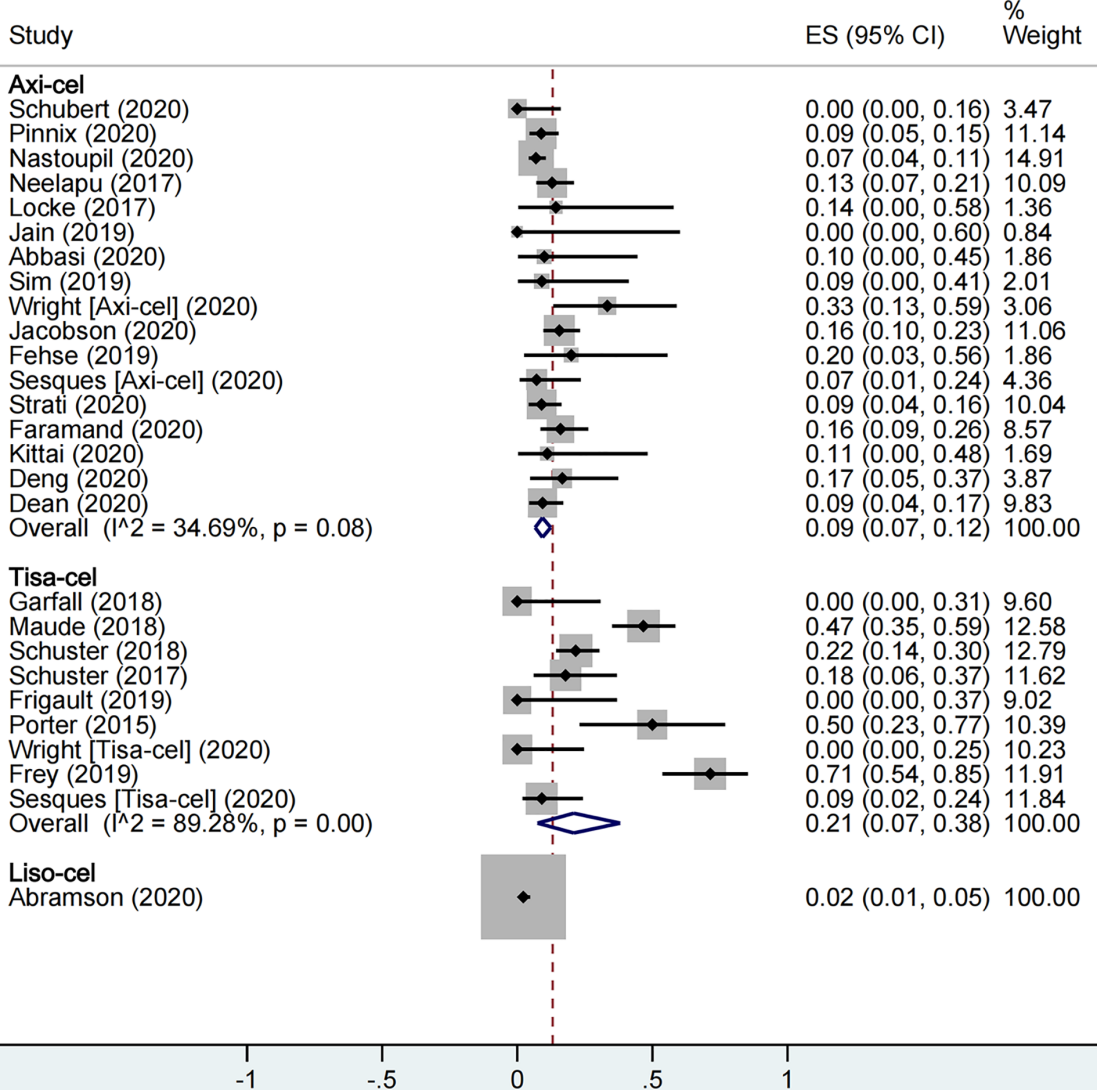
The forest plot of total severe cytokine release syndrome rate of each product.

With respect to safety, a total of 1,860 patients were included for CRS rate evaluation. The proportion of patients with severe CRS among all patients was 13% (95% CI: 0.09–0.19; I^2^ = 86.7%, P < 0.01), and the proportion was 9% (95% CI: 0.07–0.12; I^2^ = 34.7%, P = 0.08), 21% (95% CI: 0.07–0.38; I^2^ = 89.3%, P < 0.01), and 2% (95% CI: 0.01–0.05) in axi-cel, tisa-cel, and liso-cel groups, respectively (**Figure 4**). It could be observed that the tisa-cel group showed significant heterogeneity.

Previous studies have shown that the Penn scale tends to upgrade toxicity relative to other systems (52–54); thus, we conducted a subgroup analysis based on whether the Penn scale was used considering that majority of tisa-cel studies used the Penn scale. The proportion of patients with severe CRS in tisa-cel group with Penn scale and non-Penn scale were 32% (95% CI: 0.14–0.53; I^2^ = 90.3%, P < 0.01) and 4% (95% CI: 0.00–0.13; I^2^ = 0.0%, P = 0.51), and significant heterogeneity still appeared in groups using the Penn scale (**Figure S1**). Furthermore, we observed that groups using the Penn scale included all ALL studies and part of lymphoma studies; accordingly, we conducted a further subgroup analysis and observed that severe CRS rate was 55% (95% CI: 0.45–0.64; I^2^ = 0.0%) in the ALL group and 19% (95% CI: 0.06–0.36; I^2^ = 74.9%, P = 0.01) in the lymphoma group (**Figure S2**).

**Figure 5:**
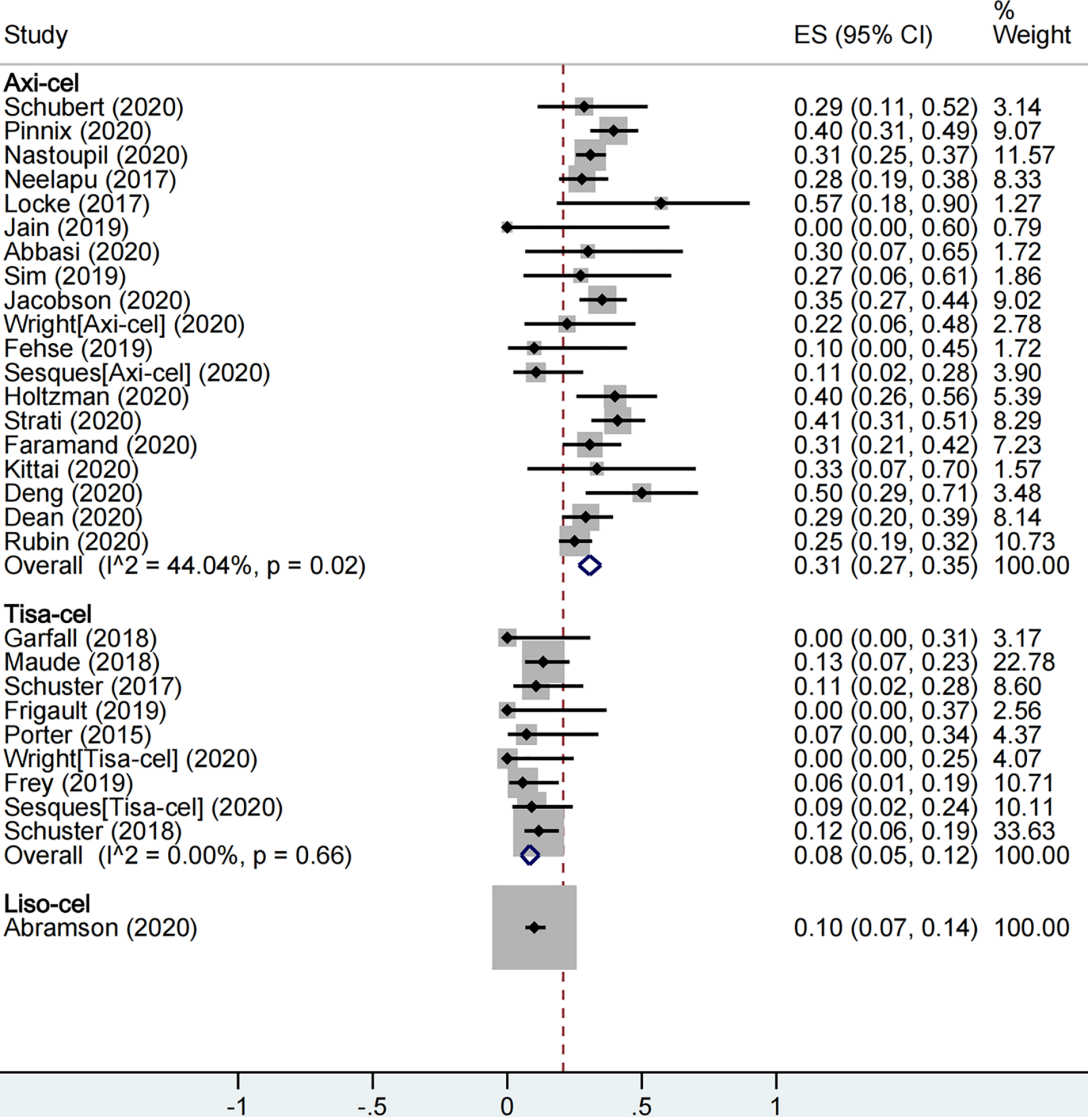
The forest plot of total severe immune effector cell-associated neurotoxicity syndrome rate of each product.

A total of 2,079 patients were included for ICANS evaluation. The overall proportion of patients with severe ICANS among all was 22% (95% CI: 0.17–0.27; I^2^ = 81.9%, P < 0.01), and the proportion was 31% (95% CI: 0.27–0.35; I^2^ = 44.0%, P = 0.02), 8% (95% CI: 0.05–0.12; I^2^ = 0.0%, P = 0.66), and 10% (95% CI: 0.07–0.14) in axi-cel, tisa-cel, and liso-cel groups, respectively (**Figure 5**).

### Subgroup Analysis Based on the Type of Tumor

Since many of the included studies simultaneously reported more than one tumor type as listed in **Table 2**, therefore, we divided them into different groups based on the type of tumor for analysis. For studies that did not report efficacy and safety results by type of tumor, we grouped them by their major tumor type (19, 26, 27, 43–47, 50). Case series that involved fewer than four patients after regrouping were excluded from analysis (**Table 3**).

**Table 3:** The results of performed meta-analysis in subgroups of product and tumor.

Subgroup	ORR, (95% CI)				CRR, (95% CI)			
	tisa-cel	axi-cel	liso-cel	Overall	tisa-cel	axi-cel	liso-cel	Overall
**All patients**	69% (.58–.79)	77% (.71–.82)	73% (.67–.78)	73% (.68–.77)	57% (.41–.72)	52% (.46–.58)	53% (.47–.59)	54% (.48–.59)
**DLBCL**	53% (.44–.61)	75% (.67–.83)	72% (.65–.78)	70% (.63–.76)	40% (.32–.49)	52% (.44–.60)	52% (.45–.59)	50% (.45–.56)
**FL/tFL**	86% (.64–1.00)	81% (.69–.90)	NA	83% (.73–.92)	73% (.48–.93)	64% (.54–.74)	NA	66% (.56–.76)
**ALL**	81% (.69–.90)	NA	NA	81% (.69–.90)	81% (.69–.90)	NA	NA	81% (.69–.90)
**Subgroup**	**CRS, (95% CI)**				**ICANS, (95% CI)**			
	**tisa-cel**	**axi-cel**	**liso-cel**	**Overall**	**tisa-cel**	**axi-cel**	**liso-cel**	**Overall**
**All patients**	21% (.07–.38)	9% (.07–.12)	2% (.01–.05)	13% (.09–.19)	8% (.05–.12)	31% (.27–.35)	10% (.07–.14)	22% (.17–.27)
**DLBCL**	8% (.01–.21)	8% (.05–.12)	NA	9% (.07–.13)	8% (.03–.13)	32% (.26–.38)	NA	25% (.19–.31)
**FL/tFL**	NA	2% (.00–.08)	NA	2% (.00–.08)	NA	31% (.18–.46)	NA	31% (.18–.46)
**ALL**	55% (.45–.64)	NA	NA	55% (.45–.64)	11% (.05–.17)	NA	NA	11% (.05–.17)

ORR, overall response rate; CRR, complete response rate; CRS, cytokine release syndrome; ICANS, immune effector cell-associated neurotoxicity syndrome; DLBCL, diffuse large B cell lymphoma; FL/tFL, follicular lymphoma or transformed follicular lymphoma; ALL, acute lymphoblastic leukemia; NA, not applicable.

#### Diffuse Large B Cell Lymphoma

**Figure 6:**
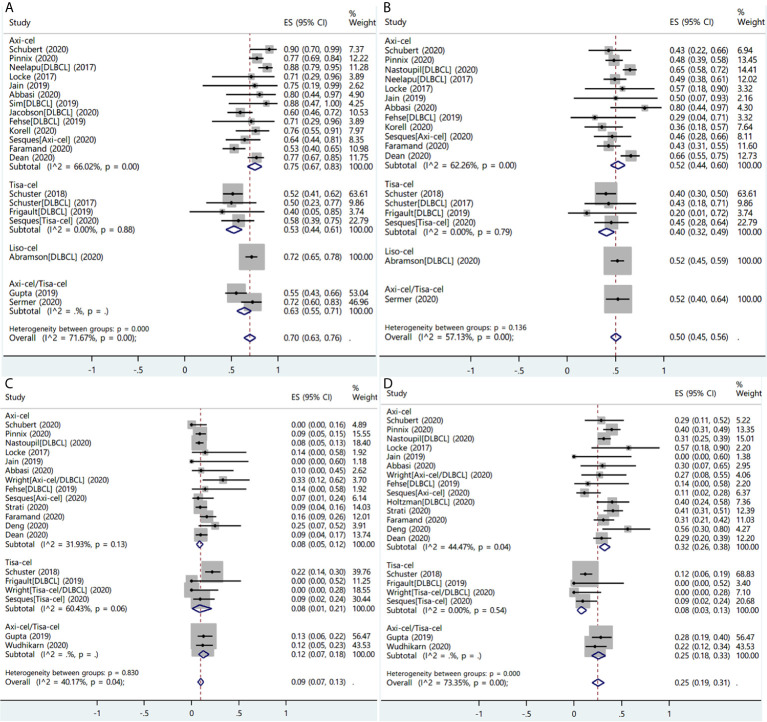
The forest plots of pooled results in patients with diffuse large B-cell lymphoma. **(A)** The forest plot of overall response rate of each product. **(B)** The forest plot of complete response rate of each product. **(C)** The forest plot of severe cytokine release syndrome rate of each product. **(D)** The forest plot of severe immune effector cell-associated neurotoxicity syndrome rate of each product.

A total of 26 studies reported the efficacy and/or safety of the three products in the treatment of DLBCL (16, 17, 19, 22, 23, 26–31, 34–36, 38–47, 49, 50). The ORR of the three products for DLBCL was 70% (95% CI: 0.63–0.76; I^2^ = 71.7%, P < 0.01), and response rate was 75% (95% CI: 0.67–0.83; I^2^ = 66.0%, P < 0.01), 53% (95% CI: 0.44–0.61; I^2^ = 0.0%, P = 0.88), and 72% (95% CI: 0.65–0.78) in axi-cel, tisa-cel, and liso-cel groups, respectively (**Figure 6A**). The CRR was 50% (95% CI: 0.45–0.56; I^2^ = 57.1%, P < 0.01) in all patients, whereas individual complete response rate was 52% (95% CI: 0.44–0.60; I^2^ = 62.3%, P < 0.01), 40% (95% CI: 0.32–0.49; I^2^ = 0.00%, P = 0.79), and 52% (95% CI: 0.45–0.59) in axi-cel, tisa-cel, and liso-cel groups, respectively (**Figure 6B**).

The proportion of patients with severe CRS among all was 9% (95% CI: 0.07–0.13; I^2^ = 40.2%, P = 0.04), and the respective rates of severe CRS for the patients with axi-cel and tisa-cel were 8% (95% CI: 0.05–0.12; I^2^ = 31.9%, P = 0.13) and 8% (95% CI: 0.01–0.21; I^2^ = 60.4%, P = 0.06) (**Figure 6C**). The study on liso-cel did not report the safety outcomes according to disease type.

Subgroup analysis was performed in tisa-cel group according to whether the Penn scale was used; the findings revealed that rates of severe CRS were 18% (95% CI: 0.11–0.26; I^2^ = 0.0%) in the group using the Penn scale and 5% (95% CI: 0.00–0.17; I^2^ = 0.0%) in the group using non-Penn scale (**Figure S3**).

The proportion of patients with severe ICANS among all was 25% (95% CI: 0.19–0.31; I^2^ = 73.4%, P < 0.01), and the respective ICANS rates for the patients with axi-cel and tisa-cel were 32% (95% CI: 0.26–0.38; I^2^ = 44.5%, P = 0.04) and 8% (95% CI: 0.03–0.13; I^2^ = 0.0%, P = 0.54) (**Figure 6D**).

#### Follicular Lymphoma or Transformed Follicular Lymphoma

**Figure 7:**
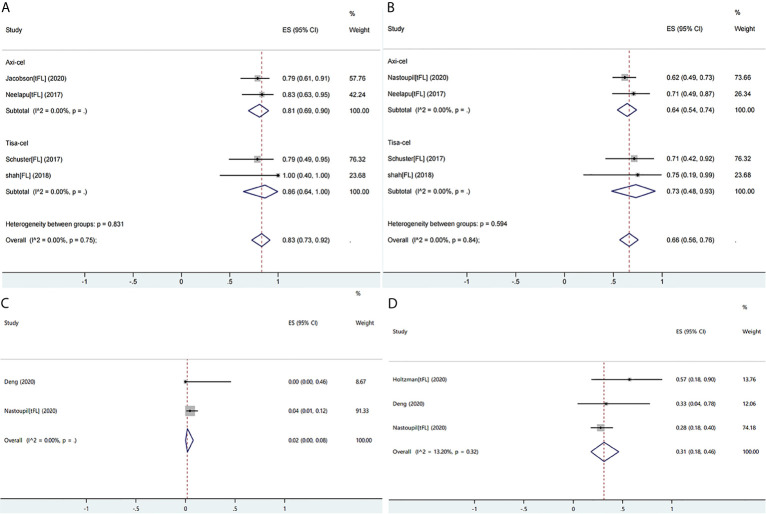
The forest plots of pooled results in patients with follicular lymphoma or transformed follicular lymphoma. **(A)** The forest plot of overall response rate of each product. **(B)** The forest plot of complete response rate of each product. **(C)** The forest plot of severe cytokine release syndrome rate of axi-cel. **(D)** The forest plot of severe immune effector cell-associated neurotoxicity syndrome rate of axi-cel.

Ten studies reported the efficacy or safety of the three products in the treatment of FL/tFL (16, 19, 28, 34, 36, 38–40, 45, 49). The ORR of the patients with FL/tFL was 83% (95% CI: 0.73–0.92; I^2^ = 0.0%, P = 0.75) and CRRs of the patients undergoing treatment with axi-cel and tisa-cel were 81% (95% CI: 0.69–0.90 I^2^ = 0.0%) and 86% (95% CI: 0.64–1.00 I^2^ = 0.0%), respectively (**Figure 7A**). The CRR of the patients with FL/tFL was 66% (95% CI: 0.56–0.76; I^2^ = 0.0%, P = 0.84), and CRRs of the patients undergoing treatment with axi-cel and tisa-cel were 64% (95% CI: 0.54–0.74; I^2^ = 0.0%) and 73% (95% CI: 0.48–0.93, I^2^ = 0.0%), respectively (**Figure 7B**). The study on liso-cel included three patients with grade 3B FL, two patients among them achieved CR and maintained it for more than one year, but the study did not report the safety events.

Three studies on axi-cel that reported ICANS or CRS events were eligible for meta-analysis, and the rates of severe CRS and ICANS were 2% (95% CI: 0.00–0.08; I^2^ = 0.0%) and 31% (95% CI: 0.18–0.46; I^2^ = 13.2%, P = 0.32), respectively (**Figures 7C, D** **)**. The severe ICANS rate in patients with tisa-cel was also acceptable, and no associated death was reported, but the available data did not support a meta-analysis.

#### Primary Mediastinal B Cell Lymphoma

Seven studies reported efficacy and safety of the three products in the patients with PMBCL, but the sample size was insufficient for their inclusion in a meta-analysis (16, 28, 35, 40, 41, 45, 49). Two real-world studies on axi-cel reported that the ORR, CRR, and rates of CRS and ICANS of the patients with PMBCL were 62% (95% CI: 0.24–0.91), 58% (95% CI: 0.33–0.80), 5% (95% CI: 0.00–0.26), and 37% (95% CI: 0.16–0.62), respectively (28, 40). A study on tisa-cel that included one patient with PMBCL with central nervous system (CNS) involvement indicated that the patient was showing ongoing response at day 90 and developed only grade 1 CRS and no ICANS (35). The study on liso-cel indicated that the ORR and CRR of the patients with PMBCL were 79% (95% CI: 0.49–0.95) and 50% (95% CI: 0.23–0.77), respectively; however, the study did not report the safety events (16).

#### High Grade B Cell Lymphoma

Three studies (each on axi-cel, tisa-cel, and liso-cel) were included to evaluate the efficacy and safety of the patients with HGBCL (16, 35, 40). The ORR and CRR of patients undergoing treatment with liso-cel were 76% (95% CI: 0.58–0.89) and 61% (95% CI: 0.42–0.77), respectively (16). The ORR was 88% (95% CI: 0.64–0.99) in the study on axi-cel, and the CRR was not reported (40). The study on tisa-cel included two patients with HGBCL with CNS involvement, one of them achieved CR, and disease progression was observed in the other patient who later experienced grade 1 CRS (35). Both axi-cel and liso-cel studies did not report the safety events.

#### Acute Lymphoblastic Leukemia and Chronic Lymphocytic Leukemia

**Figure 8:**
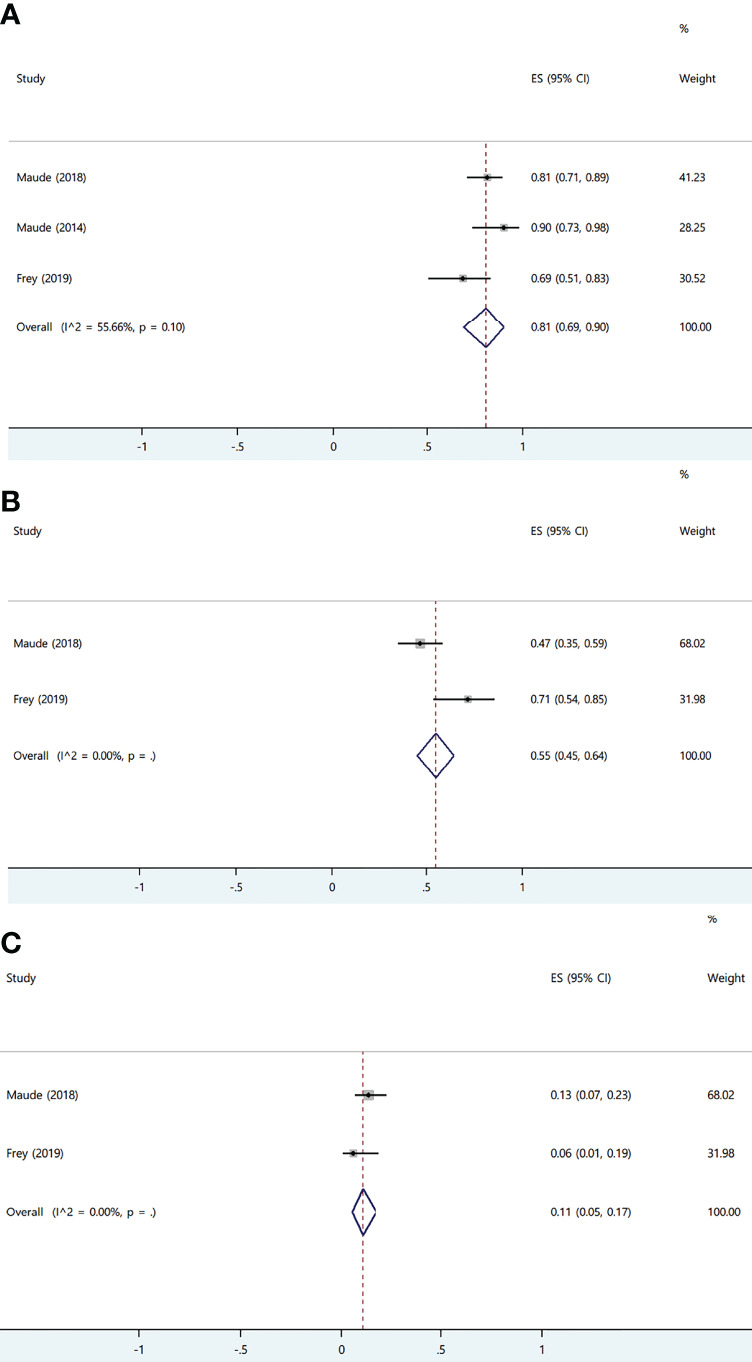
The forest plots of pooled results in patients with acute lymphoblastic leukemia. **(A)** The forest plot of complete response rate of tisa-cel. **(B)** The forest plot of severe cytokine release syndrome rate of tisa-cel. **(C)** The forest plot of severe immune effector cell-associated neurotoxicity syndrome rate of tisa-cel.

Three studies involving 135 patients reported ALL, and one study involving 14 patients reported CLL; all patients received only tisa-cel because the other two products were not approved for ALL. The CRR of the patients with ALL was 81% (95% CI: 0.69–0.90; I^2^ = 55.7%, P = 0.10). Moreover, pediatric patients showed higher CRR than that of adult patients (84 *vs.* 69%, respectively) (**Figure 8A**) (24, 25, 33).

The ORR and CRR of the patients with CLL were 57% (95% CI: 0.29–0.82) and 29% (95% CI: 0.08–0.58), respectively, and no relapse occurred in patients achieving CR with median duration of response of 40 months (range: 21–53 months) (37).

With respect to safety, the patients with ALL and CLL showed a high rate of severe CRS (ALL: 55%; 95% CI: 0.45–0.64; I^2^ = 0.0%; CLL: 50%; 95% CI: 0.23–0.77) (**Figure 8B**). High rate of CRS may only be partly attributable to the Penn scale because CRS had been observed as the most prominent and serious adverse effect for all studies on ALL, and respectively 27 and 47% of total patients in two ALL trials which were mainly pediatric patients and 29% of the patients with CLL were admitted to the intensive care unit; moreover, 9% of total patients developed grade 5 CRS in the ALL trial on adult patients (24, 25, 33, 37). Adult patients with ALL were more likely to develop grade ≥3 CRS than pediatric patients (71 *vs* 47%, respectively). The rate of severe ICANS in patients with ALL was 11% (95% CI: 0.05–0.17; I^2^ = 0.0%) and was similar in pediatric and adult cohorts (**Figure 8C**), and it was 7% (95% CI: 0.00–0.34) in patients with CLL. Neurological events typically occurred during occurrence of CRS or shortly after its resolution and were self-limited (24, 33, 37).

One study involving adult patients investigated the effects of dose levels and schedule of tisa-cel on its efficacy and safety; the results demonstrated that the high-dose fractionated cohort had superior safety and efficacy profiles compared to the low-dose cohort and the high-dose single infusion cohort; however, the other two studies did not observe apparent relationship between the dose and efficacy and/or toxicity of tisa-cel (24, 25, 33). Because the latter two studies did not set up a split dosing cohort and the patients were mainly pediatric and young adults, the doses of cells were not directly comparable to that of the adult trial. Therefore, the real impact of dosage and schedule of administration on unselected patients is not clear and deserve further investigation.

#### Other Tumor Types

A study involving 10 patients with MM revealed that patients receiving tisa-cel following autologous stem cell transplantation and high-dose melphalan experienced clinical benefit; eight patients among them achieved a partial response or better without occurrence of severe CRS and ICANS (32). A real-world study on axi-cel reported two patients with RS and five patients with TMZL discovered an overall response of one and four cases, respectively, but did not report the complete response and toxicity (40). In another study on axi-cel involving nine patients with RS, all patients were evaluated for an overall response and five patients among them achieved complete response; moreover, no fatal adverse effect was observed related to the treatment (48).

### Assessment of Publication Bias

**Figure 9:**
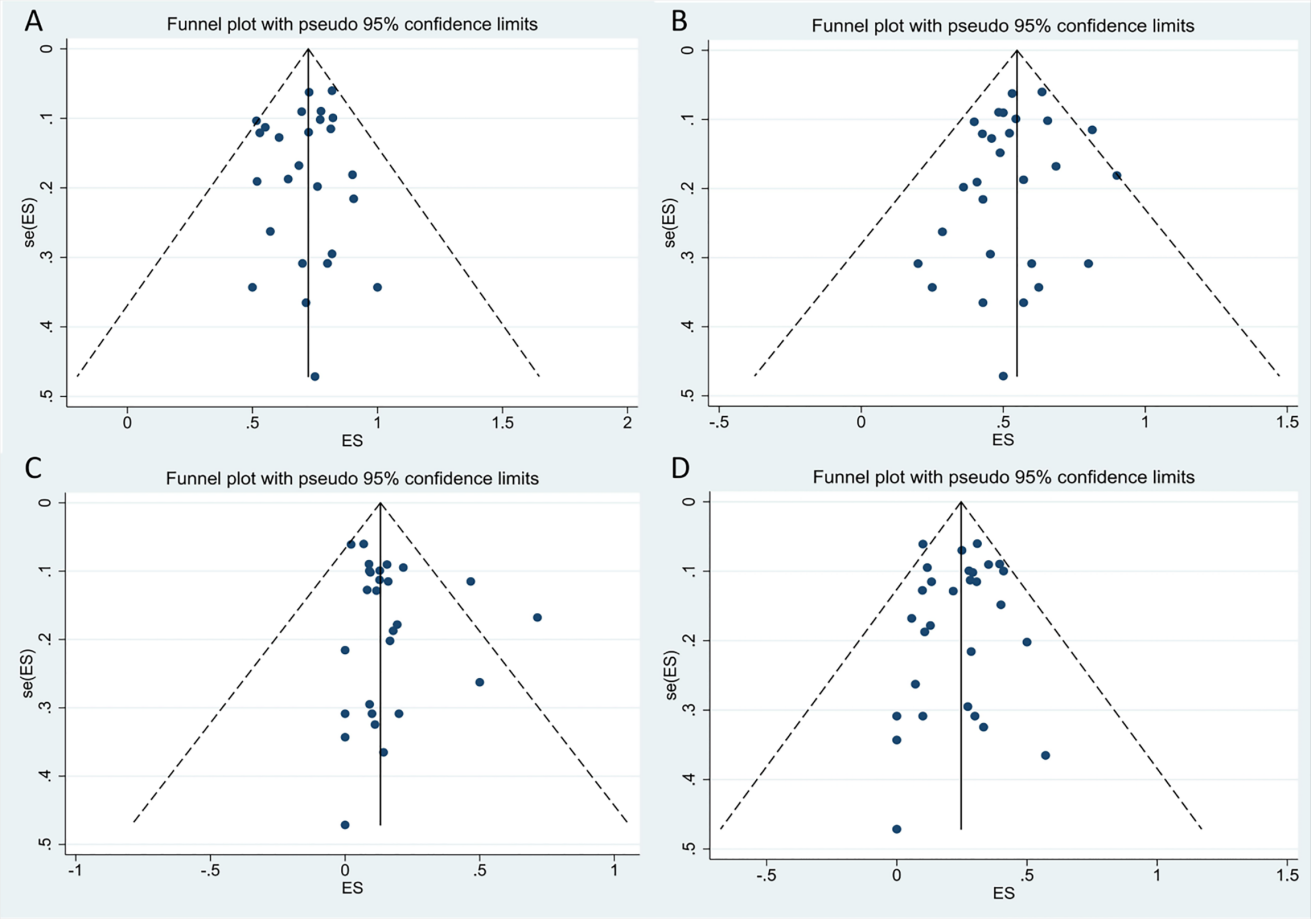
The funnel plots for included studies. **(A)** The funnel plot of total overall response rate. **(B)** The funnel plot of total complete response rate. **(C)** The funnel plot of total cytokine release syndrome rate. **(D)** The funnel plot of total immune effector cell-associated neurotoxicity syndrome rate.

We performed a funnel-plot analysis for the ORR, CRR, and rates of severe CRS and ICANS of all studies, which showed good symmetry (**Figure 9**). Egger’s and Begg’s tests were also performed, and all P-values obtained were greater than 0.5 (**Table 4**), suggesting absence of significant publication bias.

**Table 4:** Begg’s and Egger’s tests of ORR, CRR, CRS and ICANS in all included studies.

	ORR	CRR	CRS	ICAMS
Begg’s test	0.693	0.721	0.593	0.441
Egger’s test	0.437	0.290	0.151	0.713

ORR, overall response rate; CRR, complete response rate; CRS, cytokine release syndrome; ICANS, immune effector cell-associated neurotoxicity syndrome.

### Sensitivity Analysis

We used random-effects model and fixed-effects model to analyze the stability of the results in all analysis, and the two results showed a good stability in all cohorts. In the cohort with more than 75% heterogeneity, we performed subgroup analysis based on the possible causes of heterogeneity, which had been shown in the *Results* section.

### Real-World Performance of the Products

A real-world study involving 122 patients with DLBCL as the main tumor type along with tFL, HGBCL, and PMBCL observed that axi-cel had comparable overall efficacy and safety between clinical and trial settings (ORR: 70 *vs.* 68%, P = 0.25; CRR: 63 *vs.* 42%, P = 0.016; severe CRS rate: 15 *vs.* 16%; P = 0.83; severe ICANS rate: 35 *vs.* 36%; P = 0.81) in ZUMA-1 eligible and ineligible groups, respectively, although the CRR and duration of response were more favorable in patients of ZUMA-1 eligible group (40). In addition, another real-world study including 298 patients undergoing standard-of-care axi-cel treatment showed consistent results (ORR: not provided; CRR: 69 *vs.* 56%, P = 0.02; severe CRS rate: 5 *vs.* 10%; P = 0.10; severe ICANS rate: 28 *vs.* 36%; P = 0.18) in patients without *vs.* with comorbidities indicated in ZUMA-1 exclusion criteria, respectively, while the latter had shorter progression-free survival (PFS) and overall survival (OS) (28).

Of note, regarding the concerns for the inconsistency of bridging therapy in the pivotal trials, there have been two studies reported on axi-cel and one study on both axi-cel and tisa-cel that evaluated the impact of bridging therapy on the efficacy and safety of patients with R/R LBCL. Although bridging therapy is needed in patients who tended to have a higher tumor burden, the results were not significantly different between the patients with bridging therapy and non-bridging therapy; radiation therapy had been safely administered as a bridging therapy and resulted in a superior efficacy outcome (27, 36, 39).

In addition, a study which included patients with DLBCL involving CNS disease, HIV, and active HBV also provided the evidence of efficacy and safety of axi-cel in the real-world setting since these patients with severe comorbidities were excluded from the pivotal clinical trial (31). Another study that included eight patients with secondary CNS involvement LBCL receiving tisa-cel did not report severe ICANS; the findings are suggestive of potential of CAR T-cell product treatment for the patients with CNS involvement (35).

## Discussion

Our study demonstrated that the three CAR-T cell products were mainly used to treat patients with DLBCL, FL/tFL, ALL/CLL, PMBCL, and HGBCL along with other B-cell lymphomas such as MM, RS, and TMZL. Overall, the results showed promising efficacy and safety of all three products in all histological types; however, efficacy of liso-cel requires further validation from real-world data.

According to our pooled result, axi-cel showed an increased response rate in patients with lymphoma except tFL/FL as compared to tisa-cel. However, comparative efficacy of different products should be determined by randomized controlled trials in the future owing to the presence of significant heterogeneity between patients of different studies. For example, compared with JULIET trial, bridging therapy was not allowed in ZUMA-1 trial, resulting in likely exclusion of a group of patients with high tumor burden or those who are in need of emergent therapy. The ORR and CRR (77 and 52%, respectively) in patients treated with axi-cel were similar to those in the ZUMA-1 trial (ORR: 82%; CR: 54%); moreover, the ORR and CRR (57 and 44%, respectively) in patients with large B cell lymphoma and undergoing treatment with tisa-cel were similar to those in the JULIET trial (ORR: 52%; CR: 40%). In addition, we observed a favorable efficacy and manageable safety in patients with pathological subtypes that were not included in pivotal trials, such as HGBCL, MM, and RS (32, 48). However, due to the absence of large sample studies, treatment of patients with these subtypes should be monitored carefully. Another two CAR-T cell products recently approved by the FDA, brexucabtagene autoleucel and idecabtagene vicleucel, have shown excellent efficacy in R/R mantle cell lymphoma (ORR: 93%, CR: 67%) and multiple myeloma (ORR: 73%, CR: 33%), respectively. Therefore, these two CAR-T cell products should be the preferred treatment alternative in these two subtypes (55, 56).

The differences observed between safety profiles of axi-cel and tisa-cel in pivotal trials remained consistent in this analysis. Axi-cel tended to be associated with a higher rate of severe ICANS than tisa-cel (31 *vs.* 8%, respectively), possibly due to its CD28 co-stimulation domain. Therefore, patients receiving axi-cel who are at high risk of neurotoxicity, which may include preexisting endothelial activation, such as a high angiopoietin 2:angiopoietin 1 ratio and von Willebrand factor concentration in serum or other characteristics that are considered to increase the risk of neurotoxicity after CAR-T cell infusion, should be certainly monitored for serum biomarkers including interleukin (IL)-2, IL-6, IL-15, and IL-2Rα, ferritin, granulocyte–macrophage colony-stimulating factor, and other markers significantly associated with severe ICANS (19, 57). When grade 1–2 neurotoxicity occurs, corticosteroids are the preferred treatment to prevent exacerbation, and anti-IL-6 therapy is recommended in patients with concurrent CRS. In addition, the use of levetiracetam and phenobarbital to prevent seizures is important (3).

Notably, subgroup analysis indicated that higher rate of severe CRS of tisa-cel in patients with LBCL may be attributed to using the Penn scale (19% in the Penn scale group *vs.* 4% in non-Penn scale group). However, patients with ALL or CLL were at a high risk of developing severe CRS and requiring intensive care; therefore, medical staff need to closely monitor body temperature of the patient and levels of IL-6, C-reactive protein, interferon-gamma, and ferritin, and other predictive cytokines after tisa-cel infusion, especially for patients with high tumor burden. Tocilizumab can be considered as an early treatment immediately after appearance of elevation of temperature or biomarkers or low-grade CRS symptoms to prevent further exacerbation (37). The use of corticosteroids and anti-IL-6 therapy did not appear to affect the efficacy of CAR-T cells, and supportive care is also important (3, 19, 40).

In addition, CAR-T cell therapy, unlike conventional treatment, uses genetically modified cells; hence the success rate and manufacturing speed of the three products also need to be considered. In the JULIET trial, most patients discontinued participation due to disease progression; 7% of the enrolled patients discontinued due to manufacturing failure of tisa-cel and 10% due to death. In the TRANSCEND NHL 001 trial, 7% of patients received non-conforming products, two patients experienced manufacturing failure of liso-cel, and 10% of patients died before receiving liso-cel. Manufacturing time of axi-cel was approximately one week shorter than those of tisa-cel and liso-cel. In the phase 2 of ZUMA-1 trial, only 1% of patients discontinued due to manufacture failure of axi-cel and 2% of patients died before receiving axi-cel (16, 17, 19). These results reflected that the studied population is at high-risk; therefore, properties of the product manufacturing speed and success rate are crucial for patients with rapidly progressing disease.

In order to ensure presentation of high-quality and appropriate information is available, we excluded studies published in the form of conference abstracts when we conducted the study selection. Of note, conference abstracts published by several European registries indicated that patients with axi-cel and tisa-cel administration in the real-world setting showed poor responses, PFS, or OS, compared to the pivotal trial, while the adverse events were manageable and of similar intensity. Real-world data from institutions in Spain, Germany, France, and the United Kingdom showed that the ORR of patients receiving axi-cel and tisa-cel did not exceed 70%, and the CRR of patients except from France was approximately 30% (58–62). Similarly, the French and British institutions observed that patients were likely to have a higher frequency of rapid relapses and a shorter survival period than expected (58, 59, 62), Poorer outcomes may be related to greater proportion of patients with advanced stage, refractory to previous treatments, or multiple number of previous lines of treatment. In addition, although the eligible studies included a large proportion of real-world studies, including those by Nastoupil et al. and Jacobson et al., however, the intention-to-treat (ITT) analysis was not strictly implemented. They performed a modified ITT analysis (included all patients who had received CAR-T cells) or used patients with T cells collected as an ITT set, which might have overestimated effectiveness because some patients with worse conditions were excluded. In the JULIET trial, patients achieved an ORR of only 34% when all the enrolled patients were included in the ITT set (17). The most common factors related to patient discontinuation of CAR-T infusions are disease progression and death; in particular, the median time from leukapheresis to CAR T-cell infusion is longer in the real-world practice. Progression of the disease during this interval may impact the efficacy of subsequent CAR-T treatment. Bridging patients with intention to treat from leukapheresis to CAR-T cell infusion in the real world remains a challenge.

Our study has some limitations. First, the CRS and ICANS grading scales used in the included studies were not absolutely consistent. Although we analyzed the Penn scale, due to the lack of data to further analyze the non-Penn scales, it is unknown whether other scales could still confound the result. It is necessary to use a unified toxicity assessment scale in future studies. Second, all patients included in the study actually received CAR-T infusion, and no ITT analysis was performed. Therefore, the pooled results may overestimate the effectiveness of CAR-T treatment. Third, considering the quality of the research and the completeness of the information, we excluded conference abstracts without full text. Nonetheless, the data reported in these abstracts may be important to understand the performance of commercial CAR-T cells in the real world; therefore, future systematic reviews focusing on the real-world performance may need to include them. Fourth, we evaluated only two most common side effects of CAR-T cell treatment, but many patients also experienced other serious side effects such as white-cell count decreased, neutropenia, thrombocytopenia, and infection, which should be included in risk assessment in clinical setting. Fifth, we used only the ORR and CRR of the three products as efficacy endpoints, but the duration of response and median PFS of responders are of great significance as well. Furthermore, none of the included studies was a randomized controlled trial, although the risk factors that may influence the efficacy and safety outcomes of different products could be partly balanced in the real-world setting. Considering inevitable differences in the level of care and the patient baseline and lack of post-approval data on liso-cel, so our study could only provide the safety and efficacy profiles and characteristics of use for each product rather than providing a comparative result.

## Conclusion

The present meta-analysis showed promising response rate of axi-cel and tisa-cel for various B-cell malignancies; however, the high rate of severe CRS associated with tisa-cel for the treatment of leukemia and rate of ICANS with axi-cel require special attention in the clinical setting. Although liso-cel showed excellent efficacy and safety profiles, its real-world performance needs further validation. The pooled results validated consistency in efficacy and safety profiles of axi-cel and tisa-cel in real-world and clinical trial. However, in the future, it is necessary to conduct a review focusing on real-world data in the context of ITT set to further explore the factors that can affect their efficacy. In addition, some patients who did not meet eligibility criteria of pivotal trials had shown favorable results that warrant further investigation, to benefit a broader patient population.

## Data Availability Statement

The original contributions presented in the study are included in the article/**Supplementary Material**. Further inquiries can be directed to the corresponding author.

## Author Contributions

JH, ZS contributed to the conception and design of the study. RX and RH searched the database. ML and XW extracted data. JM, ZS and RX conducted data analysis. XW, JM, ZS and ML wrote the manuscript. All authors contributed to manuscript revision, read, and approved the submitted version.

## Conflict of Interest

The authors declare that the research was conducted in the absence of any commercial or financial relationships that could be construed as a potential conflict of interest.

## Publisher’s Note

All claims expressed in this article are solely those of the authors and do not necessarily represent those of their affiliated organizations, or those of the publisher, the editors and the reviewers. Any product that may be evaluated in this article, or claim that may be made by its manufacturer, is not guaranteed or endorsed by the publisher.

## Footnotes

1
https://www.fda.gov/vaccines-blood-biologics/cellular-gene-therapy-products/approved-cellular-and-gene-therapy-products

## Supplementary Material

Supplementary Table 1 The quality assessment of included studies

Supplementary Figure 1 The forest plot of severe cytokine release syndrome rate of tisa-cel by Penn and non-Penn scales. **(A)** The forest plot of severe cytokine release syndrome rate according to non-Penn scale. **(B)** The forest plot of severe cytokine release syndrome rate according to Penn scale.

Supplementary Figure 2 The forest plot of severe cytokine release syndrome rate of tisa-cel with Penn scale by pathologic subtype. **(A)** The forest plot of severe cytokine release syndrome rate in lymphoma patients. **(B)** The forest plot of severe cytokine release syndrome rate in patients with acute lymphoblastic leukemia.

Supplementary Figure 3 The forest plot of severe cytokine release syndrome rate in patients with diffuse large B-cell lymphoma receiving tisa-cel infusion by Penn and non-Penn scales. **(A)** The forest plot of severe cytokine release syndrome rate according to Penn scale. **(B)** The forest plot of severe cytokine release syndrome rate according to non-Penn scale.
