# Corrigendum: Efficacy and Safety of CAR-T Cell Products Axicabtagene Ciloleucel, Tisagenlecleucel, and Lisocabtagene Maraleucel for the Treatment of Hematologic Malignancies: A Systematic Review and Meta-Analysis

**DOI:** 10.3389/fonc.2021.768128

**Published:** 2021-10-07

**Authors:** Jun Meng, XiaoQin Wu, Zhen Sun, RenDe Xun, MengSi Liu, Rui Hu, JianChao Huang

**Affiliations:** ^1^ Molecular Genetics Laboratory, Suining Central Hospital, Suining, China; ^2^ Department of Neurosurgery, The First Affiliated Hospital, University of South China, Hengyang, China; ^3^ Hengyang Medical College, University of South China, Hengyang, China

**Keywords:** chimeric antigen receptor T-cell product, CAR-T cell therapy, immunotherapy, lymphoma, leukemia, hematologic malignancy, efficacy, safety

In the original article, there were mistakes in ***Table 2*** as published. We collected data with reference to both the conference abstract version and the full-text published version of the TRANSCEND NHL 001 trial conducted by Abramson et al. (1, 2); in ***Table 2***, we showed the data from the conference abstract version, which should be changed to the full-text version. The rest of the paper, including the meta-analysis and the systematic review section, was run with data from the full-text version of the TRANSCEND NHL 001 trial and is therefore unaffected. In addition, two footnotes were ignored in ***Table 2***. The corrected ***Table 2*** appears below.

**Table 2:** Characteristics of included studies.

First Author	Year	No.	Median age (range)-yr	Histological type	CAR-T type	Efficacy evaluation	Scale	Toxicity evaluation (grade≥3)	Scale (CRS/ICANS)	Ref
Schuster	2018	111	56 (22–76)	DLBCL	tisa-cel	CR: 37/93 PR: 11/93	Lugano	CRS: 24/111 ICANS: 13/111	Penn/CTCAE 4.03, MDRA 20.1	17
Schubert	2020	21	52 (20-68)	16 DLBCL, 3 PMBCL, 1 DHL, 1 tFL	axi-cel	CR: 9/21 PR: 10/21	Lugano	CRS: 0/21 ICANS: 6/21	ASTCT/ASTCT	22
Pinnix	2020	124	60 (18-85)	95 DLBCL, 20 tFL, 9 PMBCL	axi-cel	CR: 60/124 PR: 36/124	Lugano	CRS: 11/124 ICANS: 49/124	ASTCT, CARTOX/ASTCT, CARTOX	23
Nastoupil	2020	298	60 (21-83)	203 DLBCL, 76 tFL, 19 PMBCL	axi-cel	CR: 175/275 PR: 50/175	Lugano	CRS: 19/275 ICANS: 85/275	CARTOX, Lee/CARTOX, CTCAE 4.03	24
Neelapu	2017	101	58 (23–76)	77 DLBCL, 16 tFL, 8 PMBCL	axi-cel	CR: 55/101 PR: 28/101	IWGRC	CRS: 13/101 ICANS: 28/101	Lee/CTCAE 4.03	19
Locke	2017	7	46 (29-69)	DLBCL	axi-cel	CR: 4/7 PR: 1/7	IWGRC	CRS: 1/7 ICANS: 4/7	Lee/CTCAE 4.03	25
Jain	2019	4	56 (38-66)	DLBCL	axi-cel	CR: 2/4 PR: 1/4	NP	CRS: 0/4 ICANS: 0/4	NP/NP	26
Abbasi	2020	10	66 (55–77)	DLBCL	axi-cel	CR: 8/10 PR: 0/10	NP	CRS: 1/10 ICANS: 3/10	ASTCT/ASTCT	27
Garfall	2018	10	61 (48-68)	MM	tisa-cel	CR: 6/10^†^ PR: 2/10	IMWGRC	CRS: 0/10 ICANS: 0/10	NP/NP	28
Maude	2018	75	11 (3-23)	ALL	tisa-cel	CR: 61/75 PR: 0/75	Independent scale	CRS: 35/75 ICANS: 10/75	Penn/CTCAE 4.03	29
Maude	2014	30	14 (5-60)	ALL	tisa-cel	CR: 27/30 PR: 0/30	Independent scale	CRS: 8/30^¶^ ICANS: NP	Independent scale/NP	30
Schuster	2017	28	58 (25-77)	14 DLBCL 14 FL	tisa-cel	CR: 16/28 PR: 2/28	1999 IWGRC	CRS: 5/28 ICANS: 3/28	Penn/NP	31
Frigault	2019	8	50 (17-79)	5 DLBCL, 2 HGBCL, 1 PMBCL	tisa-cel	CR: 2/8 PR: 2/8	NP	CRS: 0/8 ICANS: 0/8	Lee, ASTCT/Lee, ASTCT	32
Sim	2019	11	NP	8 DLBCL, 3 tFL,	axi-cel	CR: 5/11 PR: 4/11	Lugano	CRS: 1/11 ICANS: 3/11	CTCAE 5.0/CTCAE 5.0	33
Porter	2015	14	66 (51-78)	CLL	tisa-cel	CR: 4/14 PR: 4/14	IWG on CLL RC	CRS: 7/14 ICANS: 1/14	Penn/CTCAE 3.0	34
Shah	2018	7	NP	3 DLBCL, 4 FL	tisa-cel	CR: 3/7 PR: 2/7	Lugano	CRS: NP ICANS: NP	NP/NP	35
Wright	2020	31	NP	26 DLBCL, 5 tFL	18 axi-cel, 13 tisa-cel	CR: 11/27 PR: 3/27	Lugano	CRS: 6/31 ICANS: 4/31	Penn/NP	36
Jacobson	2020	122	62 (21-79)	57 DLBCL, 33 tFL, 17 HGBCL, 8 PMBCL, 5 TMZL, 2 RS	axi-cel	CR: 61/122 PR: 24/122	Lugano	CRS: 19/122 ICANS: 43/122	Lee/CTCAE 4.03	37
Abramson	2020	269	63 (54-70)	215 DLBCL, 36 HGBCL, 15 PMBCL, 3 FL3B	liso-cel	CR: 136/256 PR: 50/256	Lugano	CRS: 6/269 ICANS: 27/269	Lee/CTCAE 4.03	16
Fehse	2019	10	56 (24-79)	7 DLBCL, 3 PMBCL	axi-cel	CR: 2/10 PR: 5/10	NP	CRS: 2/10 ICANS: 1/10	ASTCT/ASTCT	38
Gupta	2019	78	60+-13※	DLBCL	69 axi-cel, 9 tisa-cel	CR+PR: 43/78*	NP	CRS: 10/78 ICANS: 22/78	CTCAE 5.0, Lee/CTCAE 5.0	39
Korell	2020	25	54 (20-68)	24 DLBCL, 1 PMBCL	axi-cel	CR: 9/25 PR: 10/25	Lugano	CRS: NP ICANS: NP	NP/NP	40
Frey	2019	35	34 (21-70)	ALL	tisa-cel	CR: 24/35 PR: 0/35	Independent scale	CRS: 25/35 ICANS: 2/35	Penn/CTCAE 4.03	41
Sesques	2020	61	59 (27-75)	38 DLBCL, 18 PMBCL, 4 tFL, 1 TMZL	28 axi-cel, 33 tisa-cel	CR: 28/61 PR: 9/61	Lugano	CRS: 5/61 ICANS: 6/61	ASTCT/ASTCT	42
Holtzman	2020	45	60 (26-75)	35 DLBCL, 3 PMBCL, 7 tFL	axi-cel	CR: 22/45 PR: NP	NP	CRS: NP ICANS: 18/45	NP/CTCAE 4.03	43
Strati	2020	100	60 (18-85)	LBCL (Including 77 DLBCL)	axi-cel	CR: NP PR: NP	Lugano	CRS: 9/100 ICANS:41/100	CARTOX/CARTOX	44
Faramand	2020	75	63 (23-79	50 DLBCL, 25 Transformed Indolent lymphomas	axi-cel	CR: 36/68 PR: 29/68	Lugano	CRS: 12/75 ICANS: 23/75	ASTCT/CARTOX, ASTCT, CTCAE v4.03	45
Kittai	2020	9	64 (40-77)	RS	axi-cel	CR: 8/8 PR: 5/8	Lugano	CRS: 1/9 ICANS: 3/9	ASTCT/ASTCT	46
Deng	2020	24	58 (24-74)	16 DLBCL, 6 tFL, 2 PMBCL	axi-cel	CR: NP PR: NP	NP	CRS: 4/24 ICANS: 12/24	NP/NP	47
Dean	2020	96	64 (19-79)	47 DLBCL, 15 HGBCL, 5 PMBCL, 29 NP	axi-cel	CR: 74/96 PR: 63/96	NP	CRS: 9/96 ICANS: 28/96	Lee/CTCAE 4.03	48
Sermer	2020	69	63 (19-85)	DLBCL	47 axi-cel, 22 tisa-cel	CR: 50/69 PR: 36/69	Lugano	CRS: NP ICANS: NP	NP/NP	49
Wudhikarn	2020	60	63 (20-86)	DLBCL	43 axi-cel, 17 tisa-cel	CR: NP PR: NP	NP	CRS: 7/60 ICANS: 13/60	NP/NP	50
Rubin	2020	204	60+-12^※^	Inexact^#^	axi-cel	CR: NP PR: NP	NP	CRS: NP ICANS: 51/204	NP/CTCAE 4.03	51

CR, complete response; PR, partial response; CRS, cytokine release syndrome; ICANS, immune effector cell-associated neurotoxicity syndrome; DLBCL, diffuse large B cell lymphoma; FL/tFL, follicular lymphoma or transformed follicular; PMBCL, primary mediastinal B-cell lymphoma; HGBCL, high-grade B cell lymphoma; ALL, acute lymphoblastic leukemia; CLL, chronic lymphocytic leukemia; CAR-T, chimeric antigen receptor T; TMZL, transformed marginal zone lymphoma; MM, multiple myeloma; NP, not provided; ref, reference; MDRA, Medical Dictionary for Regulatory Activities, version; CTCAE, Common Terminology Criteria for Adverse Events; RS, Richter’ s syndrome; ASTCT, American Society for Transplantation and Cellular Therapy criteria; CARTOX, CAR-T-cell-therapy-associated TOXicity; IWGRC, International Working Group Response Criteria; IMWGRC, International Myeloma Working Group response criteria; IWG on CLL RC, International Workshop Group on CLL response criteria.

Independent scale: the institution used their own criteria instead of international criteria, which can be found in original text.

^†^Very good partial response was analyzed as complete response.

^¶^The statement in original text was severe CRS, but it was not clear if it was ≥ grade 3 and therefore not included for analysis.

*No separate CR and PR numbers were provided.

^※^Mean ± standard deviation.

^#^Patients with aggressive (e.g., diffuse large B-cell lymphoma, primary mediastinal B-cell lymphoma) or indolent (e.g., follicular lymphoma, marginal zone lymphoma) histologic subtype.

In the original article, the neurotoxicity result of tisa-cel in the Primary Mediastinal B Cell Lymphoma subgroup was incorrectly stated. A correction has been made to ***Results, Primary Mediastinal B Cell Lymphoma, Paragraph 1***.

“A study on tisa-cel that included one patient with PMBCL with central nervous system (CNS) involvement indicated that the patient was showing ongoing response at day 90 and developed only grade 1 CRS and no ICANS”.

Secondly, a percentage was carelessly written incorrectly in the comparison of severe cytokine release syndrome between adult and pediatric patients with acute lymphoblastic leukemia. A correction has been made to ***Results, Acute Lymphoblastic Leukemia and Chronic Lymphocytic Leukemia, Paragraph 3***.

“Adult patients with ALL were more likely to develop grade ≥3 CRS than pediatric patients (71 *vs* 47%, respectively)”.

In addition, we carelessly wrote “tisa-cel” instead of “liso-cel” and used a wrong percentage in one place. A correction has been made to ***Discussion, Paragraph 5***.

“In the TRANSCEND NHL 001 trial, 7% of patients received non-conforming products, two patients experienced manufacturing failure of liso-cel, and 10% of patients died before receiving liso-cel.”

In the original article, there was a mistake in the legend for ***Figure 8*** as published. “tisa-cel” was carelessly written as “axi-cel”. The correct legend appears below.

**Figure 8:**
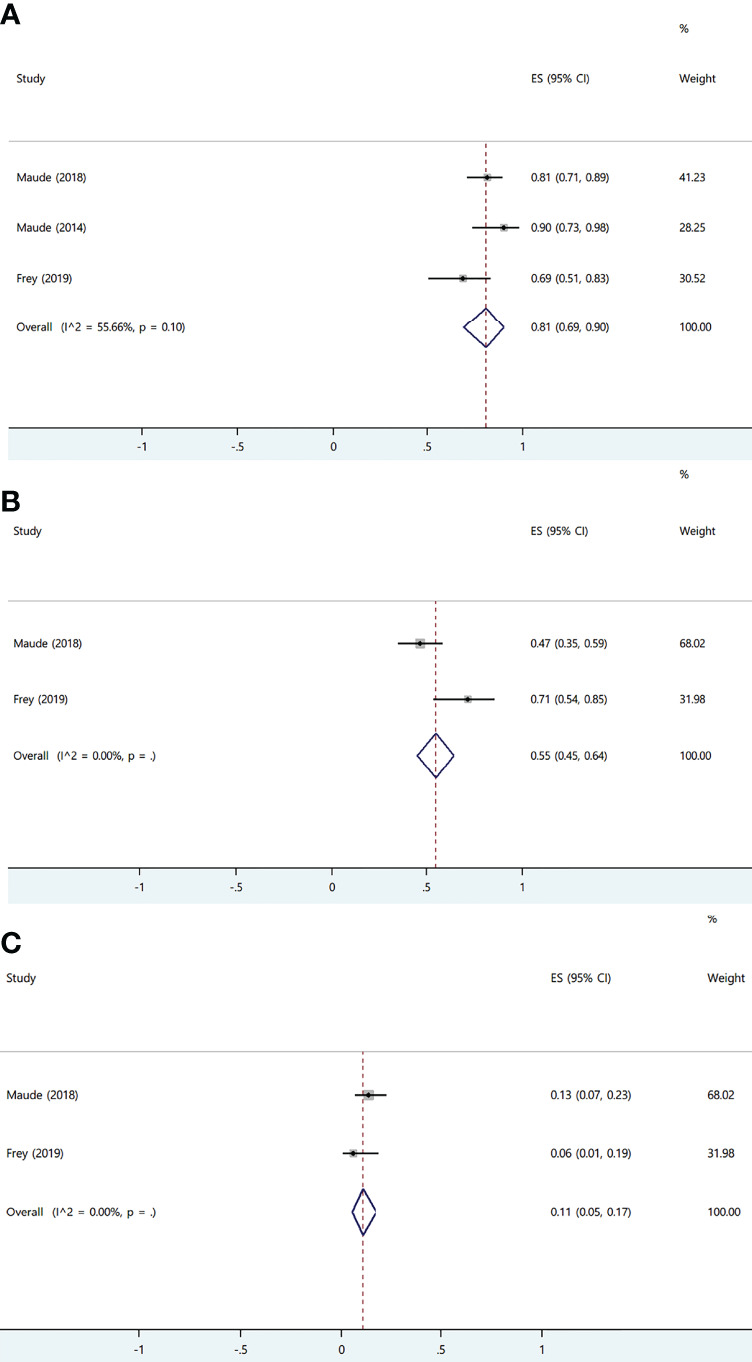
The forest plots of pooled results in patients with acute lymphoblastic leukemia. **(A)** The forest plot of complete response rate of tisa-cel. **(B)** The forest plot of severe cytokine release syndrome rate of tisa-cel. **(C)** The forest plot of severe immune effector cell-associated neurotoxicity syndrome rate of tisa -cel.

The authors apologize for these errors and state that this does not change the scientific conclusions of the article in any way. The original article has been updated.

## Publisher’s Note

All claims expressed in this article are solely those of the authors and do not necessarily represent those of their affiliated organizations, or those of the publisher, the editors and the reviewers. Any product that may be evaluated in this article, or claim that may be made by its manufacturer, is not guaranteed or endorsed by the publisher.
