# Supplementary material for: Efficacy and Safety of CAR-T Cell Products Axicabtagene Ciloleucel, Tisagenlecleucel, and Lisocabtagene Maraleucel for the Treatment of Hematologic Malignancies: A Systematic Review and Meta-Analysis
Source: Front Oncol. 2021 Jul 26;11:698607. doi: 10.3389/fonc.2021.698607 (PMC8350577; doi:10.3389/fonc.2021.698607)
Supplement: Supplementary Table 1 — The quality assessment of included studies [file DataSheet_1.docx]

| **Study** | **Selection** | | | | **Comparability** | **Outcome** | | | **Quality score** |
| --- | --- | --- | --- | --- | --- | --- | --- | --- | --- |
|  | representativeness of the exposed cohort | selection of the nonexposed cohort | Ascertainment of exposure | Demonstration that outcome of interest was not present at start of study | Comparability of cohorts on the basis of the design or analysis | Assessment of outcome | Was follow-up long enough for outcome to occur | Adequacy of follow up of cohorts |  |
| Schuster 2018 | ★ |  | ★ | ★ | ★★ | ★ | ★ | ★ | 8 |
| Schubert 2020 | ★ |  | ★ | ★ | ★ | ★ | ★ | ★ | 7 |
| Pinnix 2020 | ★ |  | ★ | ★ | ★★ | ★ | ★ | ★ | 8 |
| Nastoupil 2020 | ★ |  | ★ | ★ | ★★ | ★ | ★ | ★ | 8 |
| Neelapu 2017 | ★ |  | ★ | ★ | ★★ | ★ | ★ | ★ | 8 |
| Locke 2017 | ★ |  | ★ | ★ | ★★ | ★ | ★ | ★ | 8 |
| Jain 2019 |  |  | ★ | ★ | ★ | ★ | ★ | ★ | 6 |
| Abbasi 2020 | ★ |  | ★ | ★ | ★ | ★ | ★ | ★ | 7 |
| Garfall 2018 | ★ |  | ★ | ★ | ★ | ★ | ★ | ★ | 7 |
| Maude 2018 | ★ |  | ★ | ★ | ★★ | ★ | ★ | ★ | 8 |
| Maude 2014 | ★ |  | ★ | ★ | ★★ | ★ | ★ | ★ | 8 |
| Schuster 2017 | ★ |  | ★ | ★ | ★★ | ★ | ★ | ★ | 8 |
| Frigault 2019 |  |  | ★ | ★ | ★ | ★ | ★ | ★ | 6 |
| Sim 2019 | ★ |  | ★ | ★ | ★ | ★ | ★ | ★ | 7 |
| Porter 2015 | ★ |  | ★ | ★ | ★ | ★ | ★ | ★ | 7 |
| Shah 2018 |  |  | ★ | ★ | ★ | ★ | ★ | ★ | 6 |
| Wright 2020 | ★ |  | ★ | ★ | ★★ | ★ | ★ | ★ | 8 |
| Jacobson 2020 | ★ |  | ★ | ★ | ★★ | ★ | ★ | ★ | 8 |
| Abramson 2019 | ★ |  | ★ | ★ | ★★ | ★ | ★ | ★ | 8 |
| Fehse 2019 | ★ |  | ★ | ★ | ★ | ★ |  |  | 5 |
| Gupta 2019 | ★ |  | ★ | ★ | ★ | ★ | ★ | ★ | 7 |
| Korell 2020 | ★ |  | ★ | ★ | ★ | ★ | ★ | ★ | 7 |
| Frey 2019 | ★ |  | ★ | ★ | ★★ | ★ | ★ | ★ | 8 |
| Sesques 2020 | ★ |  | ★ | ★ | ★★ | ★ | ★ | ★ | 8 |
| Holtzman 2020 | ★ |  | ★ | ★ | ★ | ★ | ★ | ★ | 7 |
| Strati 2020 |  |  | ★ | ★ | ★ | ★ | ★ | ★ | 6 |
| Faramand 2020 | ★ |  | ★ | ★ | ★ | ★ | ★ | ★ | 7 |
| Kittai 2020 | ★ |  | ★ | ★ | ★ | ★ | ★ | ★ | 7 |
| Deng 2020 | ★ |  | ★ | ★ | ★ |  | ★ | ★ | 6 |
| Dean 2020 | ★ |  | ★ | ★ | ★★ | ★ | ★ | ★ | 8 |
| Sermer 2020 | ★ |  | ★ | ★ | ★ | ★ | ★ | ★ | 7 |
| Wudhikarn 2020 | ★ |  | ★ | ★ | ★ | ★ | ★ | ★ | 7 |
| Rubin 2020 | ★ |  | ★ | ★ | ★ | ★ |  | ★ | 6 |

**Table S1.** The quality assessment of included studies

**Search strategy of each database:**

**Pubmed:**

((Neoplasia[Title/Abstract] OR Neoplasias[Title/Abstract] OR Neoplasm[Title/Abstract] OR Tumors[Title/Abstract] OR Tumor[Title/Abstract] OR Cancer[Title/Abstract] OR Cancers[Title/Abstract] OR Malignancy[Title/Abstract] OR Malignancies[Title/Abstract] OR Malignant Neoplasms[Title/Abstract] OR Malignant Neoplasm[Title/Abstract] OR Neoplasm, Malignant[Title/Abstract] OR Neoplasms, Malignant[Title/Abstract] OR Benign Neoplasms[Title/Abstract] OR Neoplasms, Benign[Title/Abstract] OR Benign Neoplasm[Title/Abstract] OR Neoplasm, Benign[Title/Abstract]) OR ("Neoplasms"[Mesh])) AND (((((((lisocabtagene maraleucel[Title/Abstract]) OR (KYMRIAH[Title/Abstract] OR CTL019[Title/Abstract])) OR ("tisagenlecleucel" [Supplementary Concept])) OR (KTE-C19[Title/Abstract] or Yescarta[Title/Abstract])) OR ("axicabtagene ciloleucel" [Supplementary Concept]))) OR (tisagenlecleucel[Title/Abstract]))

**Web of science:**

TS= ((Neoplasms OR Neoplasia OR Neoplasias OR Neoplasm OR Tumors OR Tumor OR Cancer OR Cancers OR Malignancy OR Malignancies OR Malignant Neoplasms OR Malignant Neoplasm OR Neoplasm, Malignant OR Neoplasms, Malignant OR Benign Neoplasms OR Neoplasms, Benign OR Benign Neoplasms OR Neoplasms, Benign) AND ((Axicabtagene ciloleucel OR KTE-C19 OR Yescarta) OR (KYMRIAH OR CTL019 OR Tisagenlecleucel) OR (lisocabtagene maraleucel)))

Databases= WOS, KJD, Medline, RSCI, SCIELO

Timespan=All years

Search language=Auto

**Embase:**

('malignant neoplasm'/exp OR 'cancer':ab,ti OR 'cancers':ab,ti OR 'malignant neoplasia':ab,ti OR 'malignant neoplastic disease':ab,ti OR 'malignant tumor':ab,ti OR 'malignant tumour':ab,ti OR 'neoplasia, malignant':ab,ti OR 'tumor, malignant':ab,ti OR 'tumour, malignant') AND ('axicabtagene ciloleucel'/exp OR 'kte c19':ab,ti OR 'kte c19 car':ab,ti OR 'ktec19':ab,ti OR 'yescarta' OR 'tisagenlecleucel':ab,ti OR 'kymriah':ab,ti OR 'ctl019' OR 'lisocabtagene maraleucel'/exp OR 'jcar 017':ab,ti OR 'jcar 17':ab,ti OR 'jcar017':ab,ti OR 'jcar17':ab,ti OR 'liso-cel')

Sources: Embase, MEDLINE

**Cochrane library:**

#1 lisocabtagene maraleucel

#2 Tisagenlecleuce

#3 KYMRIAH

#4 CTL019

#5 axicabtagene ciloleucel

#6 kte c19

#7 kte c19 car

#8 ktec1

#9 yescarta

#10 #1 or #2 or #3 or #4 or #5 or #6 or #7 or #8 or #9

#11 MeSH descriptor: [Neoplasms] explode all trees

#12 (Synonyms): ab,ti,kw or (Neoplasia; Tumor): ab,ti,kw or (Neoplasias): ab,ti,kw or (Tumors): ab,ti,kw or ( Neoplasm): ab,ti,kw or (Cancer): ab,ti,kw or (Malignancy): ab,ti,kw or (Cancers): ab,ti,kw or (Malignancies): ab,ti,kw or (Malignant Neoplasms): ab,ti,kw or (Neoplasm, Malignant): ab,ti,kw or (Malignant Neoplasm): ab,ti,kw or (Neoplasms, Malignant): ab,ti,kw or (Benign Neoplasms): ab,ti,kw or (Benign Neoplasm): ab,ti,kw or (Neoplasm, Benign): ab,ti,kw or (Neoplasms, Benign): ab,ti,kw

#13 #11 or #12

#14 #10 and #13
