# Supplementary figures and images for: Efficacy and Safety of CAR-T Cell Products Axicabtagene Ciloleucel, Tisagenlecleucel, and Lisocabtagene Maraleucel for the Treatment of Hematologic Malignancies: A Systematic Review and Meta-Analysis
Source: Front Oncol. 2021 Jul 26;11:698607. doi: 10.3389/fonc.2021.698607 (PMC8350577; doi:10.3389/fonc.2021.698607)

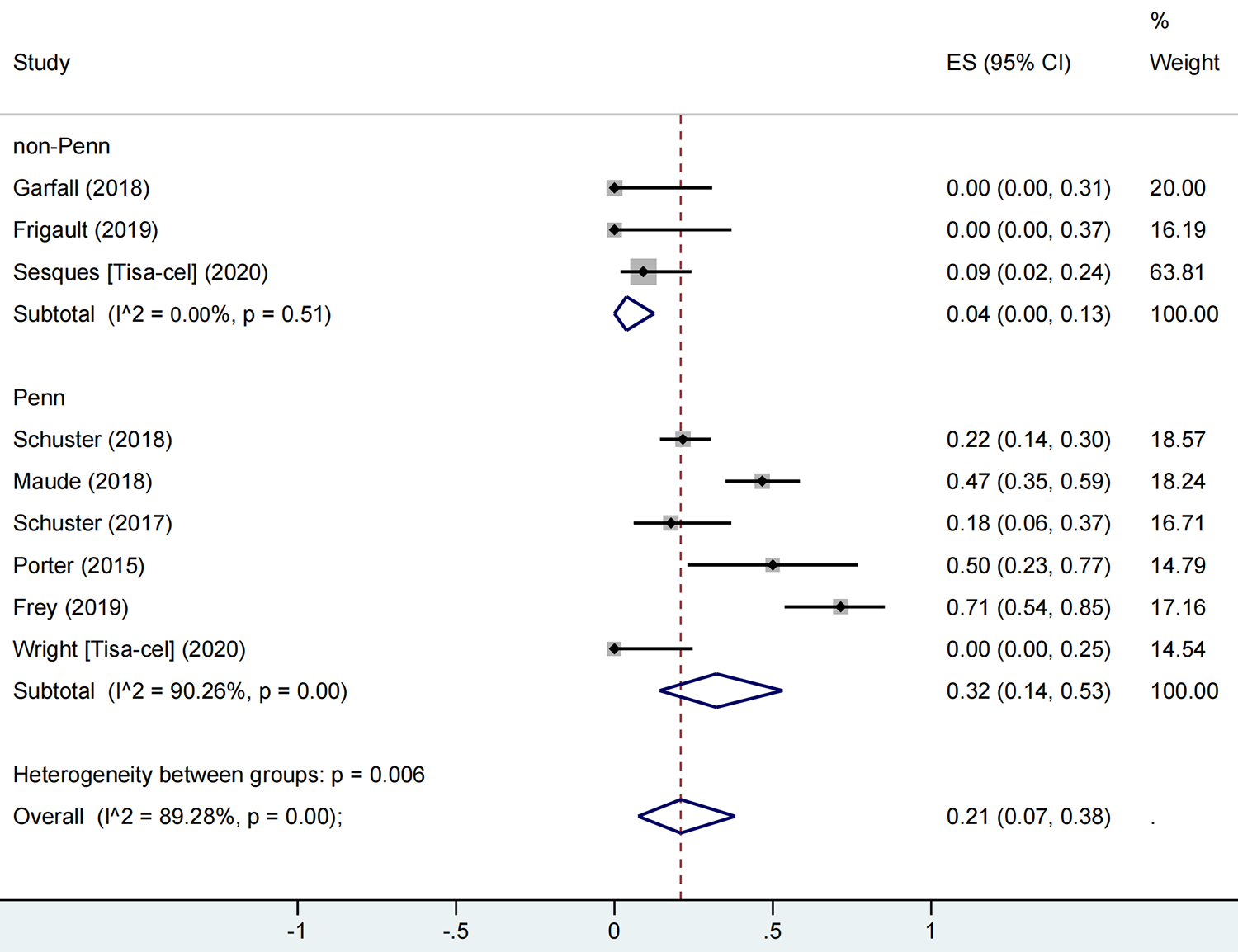

Supplement: Supplementary Figure 1 — The forest plot of severe cytokine release syndrome rate of tisa-cel by Penn and non-Penn scales. (A) The forest plot of severe cytokine release syndrome rate according to non-Penn scale. (B) The forest plot of severe cytokine release syndrome rate according to Penn scale. [file Image_1.tif]

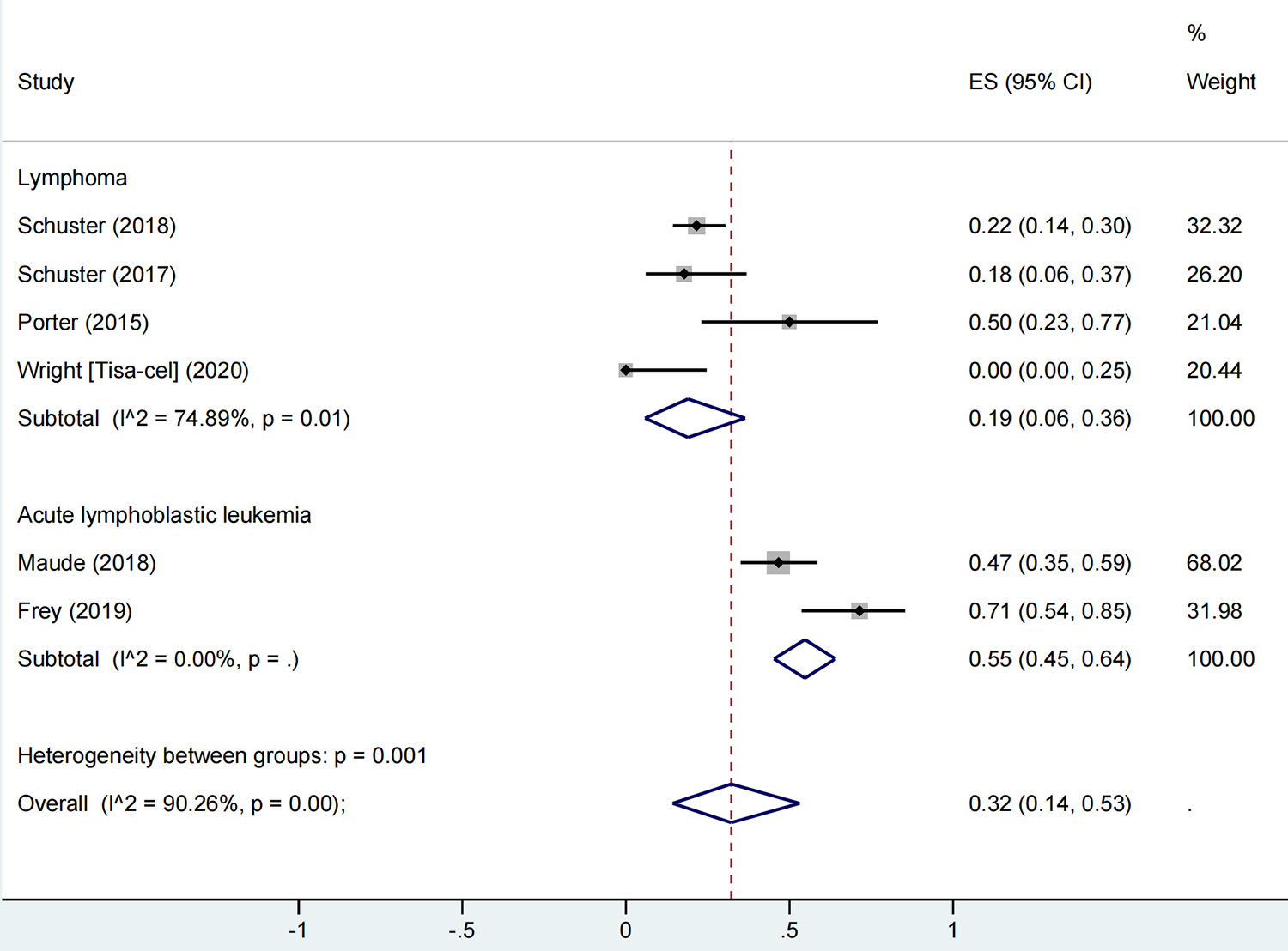

Supplement: Supplementary Figure 2 — The forest plot of severe cytokine release syndrome rate of tisa-cel with Penn scale by pathologic subtype. (A) The forest plot of severe cytokine release syndrome rate in lymphoma patients. (B) The forest plot of severe cytokine release syndrome rate in patients with acute lymphoblastic leukemia. [file Image_2.tif]

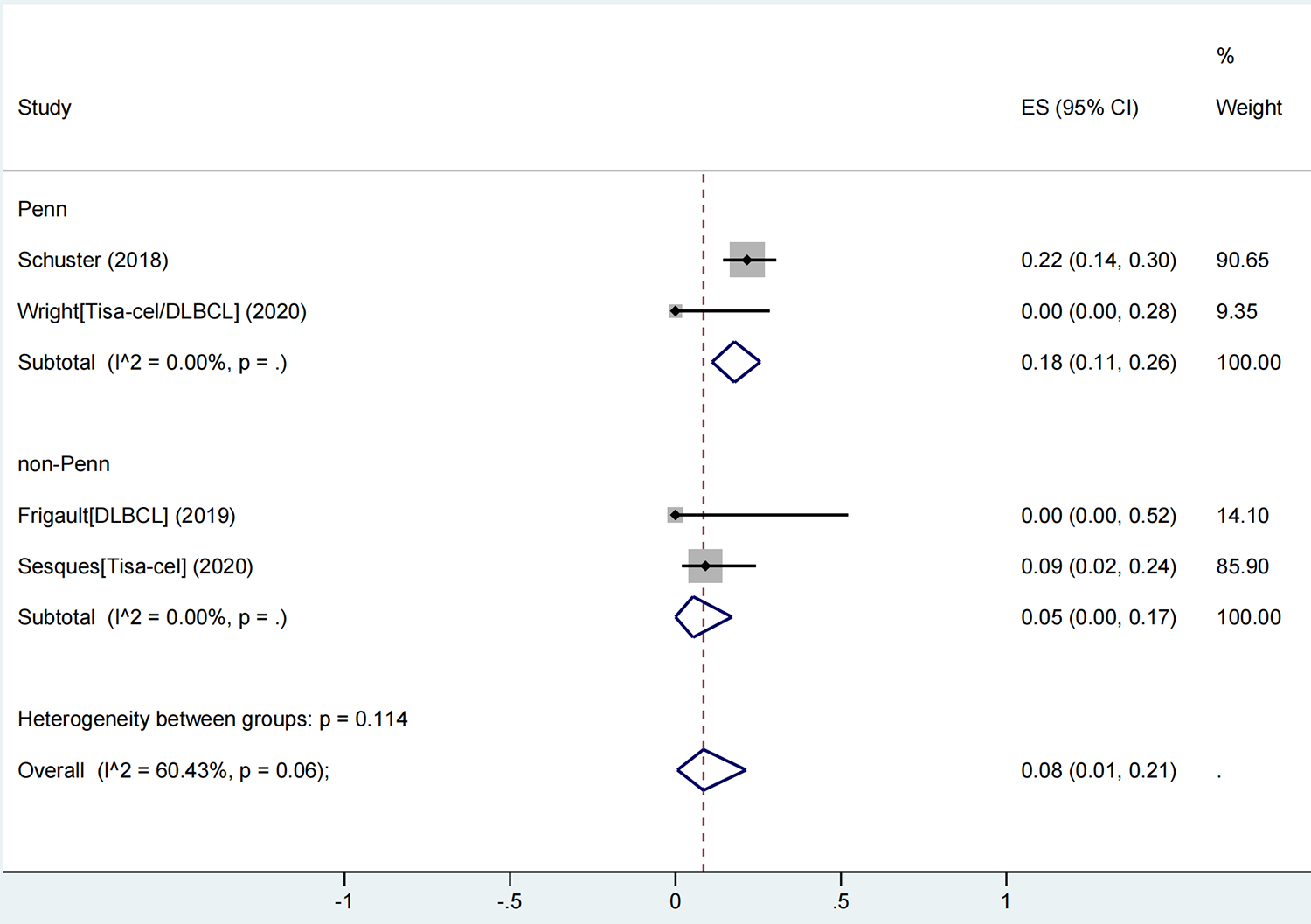

Supplement: Supplementary Figure 3 — The forest plot of severe cytokine release syndrome rate in patients with diffuse large B-cell lymphoma receiving tisa-cel infusion by Penn and non-Penn scales. (A) The forest plot of severe cytokine release syndrome rate according to Penn scale. (B) The forest plot of severe cytokine release syndrome rate according to non-Penn scale. [file Image_3.tif]
